# Pyrochar versus hydrochar: contrasting modulation of soil bacterial communities under severe nitrate-dominated acid rain stress in a *Quercus acutissima* plantation

**DOI:** 10.3389/fmicb.2026.1961661

**Published:** 2026-09-29

**Authors:** Yuanyuan Feng, Shushu Yao, Ke Yuan, Haibo Hu, Peng Cui, Yanfang Feng, Lihong Xue

**Affiliations:** 1 Co-Innovation Center for Sustainable Forestry in Southern China, College of Forestry and Grassland, Nanjing Forestry University, Nanjing, China; 2 Key Laboratory of Agro-Environment in Downstream of Yangtze Plain, National Agricultural Experiment Station for Agricultural Environment (Luhe), Ministry of Agriculture and Rural Affairs, Institute of Agricultural Resources and Environment, Jiangsu Academy of Agricultural Sciences, Nanjing, China; 3 Department of Applied Physics and Electronics, Umeå University, Umeå, Sweden

**Keywords:** bacterial community, hydrochar, predicted functional potential, pyrochar, nitrate-dominated acid rain

## Abstract

Nitrate-dominated acid rain (NAR) has become an increasingly prevalent component of contemporary atmospheric deposition and can profoundly alter soil bacterial communities in forest ecosystems. Biochar amendment has been proposed as a potential strategy for modifying soil conditions under acid deposition stress. However, the material-dependent effects of pyrochar (PC) and hydrochar (HC) on bacterial community responses under severe NAR stress remain unclear. Here, a field experiment was conducted in a *Quercus acutissima* plantation to compare the effects of PC and HC on soil bacterial community structure and predicted functional potential under simulated severe NAR stress. Compared with the NAR-only treatment, PC and HC reduced the relative abundance of Chloroflexi by 41.20 and 38.06%, respectively, and increased Actinobacteriota by 43.30 and 38.52%. LEfSe analysis revealed Chloroflexi as a phylum showing differential enrichment under NAR stress, whereas Acidobacteriota and Proteobacteria were differentially enriched in PC-NAR and HC-NAR treatments, respectively. Predicted phenotypic and functional profiles further indicated different bacterial responses to PC and HC. Exploratory association analysis suggested that PC-associated responses were more closely related to microbial biomass-associated variables, whereas HC-associated responses showed stronger relationships with soil chemical properties and microbial biomass. These findings highlight the material-dependent roles of PC and HC as carbon-based amendments and provide field-based insights into selecting biochar types for managing bacterial community responses in NAR-affected plantation soils.

## 1 Introduction

Acid rain (AR) has been a major global environmental issue since the 1980s (Hu et al., 2025). In recent decades, declines in SO_2_ emissions and persistently high nitrogen oxide (NO_x_) emissions have substantially altered the chemical composition of acid deposition (Yu et al., 2024). AR has gradually shifted from sulfuric acid-dominated AR to mixed sulfuric-nitric AR and even nitrate-dominated acid rain (NAR) (Li et al., 2021; Zhang et al., 2024). This transition is particularly pronounced in China’s Yangtze River Delta, where the SO_4_^2−^/NO_3_^−^ ratio in precipitation has continued to decline, and nitrate has made an increasing contribution to acid deposition (Zhou et al., 2022). Severe NAR imposes a higher acid load and greater reactive nitrogen (N) input than low-intensity NAR, which may intensify soil acidification, nutrient imbalance, and ecological disturbance (Li et al., 2024; Du et al., 2025). Developing effective carbon-based amendment strategies is therefore important for managing soil acidification and microbial ecological responses in NAR-affected forest soils.

Biochar amendment has been widely regarded as a promising carbon-based strategy for mitigating soil acidification and improving soil environmental quality. Its beneficial effects are generally associated with alkalinity release, ion adsorption, nutrient retention, and microhabitat modification, through which biochar may alleviate acid stress and influence soil microbial processes (Yu et al., 2021; Yang et al., 2022; An et al., 2025). Pyrochar (PC) and hydrochar (HC) are two representative biochar types. PC is typically produced by pyrolyzing biomass under oxygen-limited and high-temperature conditions. PC generally has high aromaticity, a well-developed pore structure, and a relatively large specific surface area. Such material properties can enhance the adsorption of acidic ions, improve soil microhabitats, and provide favorable microsites for microbial colonization (Yang et al., 2022; An et al., 2025). HC is commonly produced through hydrothermal carbonization of biomass under high-temperature and high-pressure conditions. Compared with PC, HC usually contains more oxygen-containing functional groups and relatively labile organic components. These characteristics may allow HC to regulate microbial processes by changing soil chemical conditions, nutrient availability, and the supply of readily utilizable carbon (Gascó et al., 2018; Zhang et al., 2024). The contrasting properties of PC and HC suggest that the two materials may remediate NAR-affected soils through different material-dependent pathways.

Soil bacterial communities are important biological indicators of soil environmental conditions because they regulate organic matter decomposition, nutrient transformation, and elemental cycling in forest ecosystems (Lladó et al., 2017). Acid deposition can disrupt these microbial processes by altering bacterial abundance, community composition, and predicted functional potential (Li et al., 2024; Shu et al., 2023). Such responses suggest that bacterial communities can sensitively reflect soil ecological changes under acid deposition stress. However, most previous studies have focused on microbial responses to acid deposition itself or to a single biochar type. Field evidence remains limited on how PC and HC differentially regulate soil chemical conditions, bacterial community responses, and predicted functional responses under severe NAR stress.

In this study, a field experiment was conducted in a *Quercus acutissima* plantation in the Yangtze River Delta to compare the responses of soil bacterial communities to PC and HC amendments under simulated severe NAR. The objectives were to: (1) evaluate the effects of PC and HC on bacterial community composition and diversity; (2) assess treatment-related changes in predicted bacterial phenotypic traits and predicted functional potential related to carbon, nitrogen, phosphorus, and sulfur cycling; and (3) explore the associations of soil chemical properties, microbial biomass, and enzyme activities with bacterial community composition and predicted functional potential under PC and HC amendments. We further hypothesized that PC and HC would regulate bacterial communities through different environmental associations, with PC effects being more related to microbial habitat modification and HC effects being more related to changes in soil chemical conditions.

## 2 Materials and methods

### 2.1 Site description

This study was conducted at the Yangtze River Delta Forest Ecosystem Long-term Observation Station in Jurong, Jiangsu Province, China (119°12′7″E, 32°7′49″N), at a mean elevation of 180 m. The site is located in the northern subtropical monsoon climate zone and is dominated by approximately 70-year-old *Q. acutissima* stands. The simulated NAR experiment was conducted from June 2021 to June 2022, during which the mean annual air temperature and annual precipitation were 15.1 °C and 1,184.3 mm, respectively. The soil at the site is classified as yellow-brown soil according to the Chinese soil genetic classification system and as Alfisols according to USDA Soil Taxonomy (Baillie, 2006). Prior to treatment application, the experimental site had the following background soil physicochemical properties: pH, 5.0; soil organic carbon (SOC), 33.5 g kg^−1^; total nitrogen (TN), 3.2 g kg^−1^; bulk density; 1.4 g cm^−3^; and soil moisture content, 14.6%.

### 2.2 Experimental design

The PC and HC used in this study were produced from *Q. acutissima* leaves collected from Xiashu Forest Farm, Nanjing, China. For PC production, air-dried *Q. acutissima* leaves were placed in covered crucibles to restrict atmospheric oxygen ingress and maintain oxygen-limited conditions during thermal treatment. The crucibles were heated in a muffle furnace from room temperature to 500 °C at a heating rate of 5 °C min^−1^ and maintained at 500 °C for 2 h. After thermal treatment, the covered crucibles were allowed to cool naturally to room temperature inside the furnace before being opened. The resulting PC was ground, passed through a 2 mm sieve, and stored in sealed containers until use. For HC production, finely ground *Q. acutissima* leaf litter (<2 mm) was subjected to hydrothermal carbonization with deionized water at a biomass-to-water ratio of 1:10 (w/v). Specifically, 600 g of dried biomass and 6 L of deionized water were loaded into a high-pressure hydrothermal reactor (BR-10 L, China). The sealed reactor was heated from room temperature to 260 °C under autogenous pressure and maintained at the target temperature for 1 h. After completion of the reaction, the reactor was allowed to cool naturally to room temperature before opening. The hydrothermal products were subsequently separated into solid and liquid fractions, and the recovered solid fraction was collected as HC without further washing. The obtained HC was dried at 70 °C to constant weight, sieved through a 2 mm mesh, and stored in sealed containers until further use.

Given the increasing contribution of nitrate to acid deposition in the Yangtze River Delta (Li et al., 2021), a severe nitrate-dominated acid rain (NAR) treatment was simulated in this study. Four treatments were established: CK, sprayed with deionized water only and not amended with biochar; NAR, sprayed with simulated severe NAR only; PC-NAR, amended with PC and sprayed with simulated severe NAR; and HC-NAR, amended with HC and sprayed with simulated severe NAR. The deionized water used for the CK treatment had a pH of approximately 6.5. For the PC-NAR and HC-NAR treatments, PC and HC, respectively, were applied once at the beginning of the experiment in June 2021 at a rate of 0.5 kg m^−2^ (5 t ha^−1^) (Cornelissen et al., 2018; Sun et al., 2024) and incorporated into the 0–10 cm soil layer. The field experiment was established using a randomized complete block design with three spatial blocks within the plantation. Treatments were randomly assigned within each block, resulting in three independent replicate plots per treatment and 12 experimental plots in total. Each plot covered an area of 3 m × 3 m and was separated from adjacent plots by a 3 m buffer zone. To minimize potential lateral transfer of the simulated NAR solution between neighboring plots, PVC barriers were installed around each plot. The PVC panels were 30 cm high and 0.5 cm thick, with 25 cm embedded in the soil and 5 cm extending above the soil surface. Soil samples were collected on 30 June 2022, approximately 2 weeks after the final simulated NAR application. In each plot, five soil cores (0–10 cm) were collected from the four corners and the center and combined into one composite sample, with each composite sample representing one independent experimental plot. Detailed procedures for soil sample processing, storage, and physicochemical analyses are provided in the Supplementary Materials.

To approximate local precipitation patterns, the monthly volume of simulated NAR was determined based on precipitation records collected at the Yangtze River Delta Forest Ecosystem Research Station from 2011 to 2021 (Zhou et al., 2022). The simulated severe NAR solution was prepared by mixing H_2_SO_4_ and HNO_3_ at an SO_4_^2−^: NO_3_^−^ molar ratio of 1:5. Following previous studies in which pH 2.5 was used as a high-acidity NAR treatment (Zhou et al., 2022), the solution was adjusted to pH 2.5 to represent severe NAR stress rather than the mean acidity of local precipitation. Beginning in June 2021, simulated NAR was uniformly applied to the corresponding plots once per month, around mid-month. The monthly simulated NAR application volume ranged from 17.89 to 106.20 L plot^−1^, with a cumulative volume of 591.56 L plot^−1^ (equivalent to 65.73 mm yr.^−1^ of water input) during the 12-month treatment period. The corresponding annual N and S inputs were 10.34 kg N ha^−1^ yr.^−1^ and 4.74 kg S ha^−1^ yr.^−1^, respectively. The nominal free H^+^ input estimated from the solution pH and cumulative spray volume normalized to a hectare basis was approximately 2.08 × 10^3^ mol H^+^ ha^−1^ yr.^−1^. Detailed monthly simulated NAR application volumes and corresponding N, S, and H^+^ inputs are provided in Supplementary Table S2.

### 2.3 Biochar characterization

The physicochemical and surface characteristics of PC and HC were analyzed before field application. Biochar pH, organic carbon content, total nitrogen content, specific surface area, and pore characteristics were determined, with the results summarized in Supplementary Table S1. Surface morphology was examined by scanning electron microscopy (SEM). Prior to SEM observation, dried biochar samples were mounted on conductive adhesive tape and coated with a thin layer of platinum to improve electrical conductivity. Surface oxygen-containing functional groups were characterized by X-ray photoelectron spectroscopy (XPS) using monochromatic Al Kα radiation. The binding energy scale was calibrated against the C1s peak at 284.8 eV. High-resolution O1s spectra were fitted using Thermo Scientific Avantage software with a Shirley-type background and mixed Gaussian-Lorentzian peak functions. The relative contribution of each O1s component was calculated based on the fitted peak area.

### 2.4 DNA extraction and amplicon sequencing

Total microbial DNA was extracted from soil samples using the E. Z. N. A.® Soil DNA Kit (Omega Bio-Tek, Norcross, GA, United States) according to the manufacturer’s instructions. The V4-V5 region of the bacterial 16S rRNA gene was amplified using specific primers with sample-specific barcodes. The PCR products were purified, quantified, and pooled at equimolar concentrations for library construction. Paired-end libraries were prepared following the Illumina standard protocol and sequenced on an Illumina NovaSeq 6000 platform by Nanjing GenePioneer Co., Ltd. The sequencing data have been deposited in the China National Center for Bioinformation (CNCB) database under BioProject accession number PRJCA063816.

Bioinformatic processing of the 16S rRNA gene amplicon sequences was performed using an ASV-based pipeline. Briefly, paired-end reads were merged using PANDAseq (version 2.11) based on the overlap between paired-end reads, with a minimum overlap length of 10 bp. Quality filtering was performed using PRINSEQ (version 0.20.4), and sequences with an average quality score below 20 or with ambiguous bases accounting for more than 5% of the sequence length were removed. The filtered sequences were processed using the DADA2 pipeline implemented in QIIME2 (version 2021.11) for primer removal, denoising, error correction, sequence merging, and chimera removal. Representative ASV sequences were assigned taxonomy using the QIIME2 classify-sklearn Naive Bayes classifier against the SILVA 138 rRNA database. Sequences not assigned to Bacteria were removed before downstream bacterial community analyses. To normalize sequencing depth, the ASV abundance table was rarefied to 24,141 sequences per sample and used for subsequent *α*-diversity and Bray-Curtis-based *β*-diversity analyses. Good’s coverage values ranged from 99.22 to 100.00% across samples.

### 2.5 Phenotype prediction of soil bacterial communities

Predicted bacterial community phenotypes were analyzed using BugBase based on the bacterial 16S rRNA gene sequencing-derived ASV abundance table. The analysis was conducted by Nanjing GenePioneer Co., Ltd. following the BugBase framework described by Ward et al. (Ward et al., 2017). Briefly, the ASV abundance table was first normalized according to microbial phylogenetic information and used as input for phenotype prediction. BugBase integrates taxonomic information from 16S rRNA gene profiles with precomputed phenotype annotations derived from reference genome resources to estimate the relative abundance of microbial taxa associated with specific community-level phenotypic traits.

For each sample, BugBase calculated the proportion of the bacterial community predicted to possess specific phenotypic potentials based on the cumulative contribution of taxa carrying the corresponding trait annotations. The predicted phenotype categories included Gram-positive, Gram-negative, biofilm-forming, potentially pathogenic, mobile-element-containing, oxygen-utilizing (aerobic, anaerobic, and facultatively anaerobic), and oxidative-stress-tolerant phenotypes.

### 2.6 Functional prediction of soil bacterial communities

Potential functional profiles of soil bacterial communities were inferred using PICRUSt2 (version 2.4.2) based on the ASV abundance table generated from 16S rRNA gene sequencing. PICRUSt2 generated Kyoto Encyclopedia of Genes and Genomes (KEGG) Orthology (KO) abundance profiles after normalization based on predicted 16S rRNA gene copy numbers. The resulting KO abundance matrix was subsequently annotated for C-, N-, P-, and S-cycling functions using the elemental-cycle annotation tool provided by Wekemo Bioincloud. KOs with zero abundance across all samples were excluded, and no additional abundance-based filtering threshold was applied. Following this procedure, 69 C-cycling, 21 N-cycling, 50 P-cycling, and 13 S-cycling KOs were retained for downstream analyses. The complete list of retained KO identifiers and corresponding functional annotations is provided in Supplementary Table S3.

### 2.7 Statistical analysis

Statistical analyses were performed using SPSS 26.0 and R software. Considering the randomized complete block design (RCBD), univariate variables, including dominant bacterial taxa, *α*-diversity indices, predicted BugBase-derived phenotypes, and PICRUSt2-derived KOs, were analyzed using linear mixed models with Treatment as a fixed effect and Block as a random effect. Tukey-adjusted pairwise comparisons based on estimated marginal means were performed when significant treatment effects were detected (*p* < 0.05). LEfSe analysis was used to identify treatment-associated bacterial taxa based on the Kruskal-Wallis test (*p* < 0.05) and an LDA score threshold of >4.0. Bacterial community composition was compared using PERMANOVA based on Bray–Curtis dissimilarity with the adonis2 function in vegan. The model included Block and Treatment as factors, with permutations restricted within blocks. Pairwise comparisons and multivariate dispersion tests (betadisper) were conducted using the same permutation framework. Similarity of amended treatments to CK was quantified using Euclidean distances to the CK centroid calculated from Bray-Curtis PCoA coordinates. Mantel tests were performed using the mantel_test function in the linkET package. Before analysis, ASV abundance data, predicted functional profiles, and environmental variables were log_10_(x + 1)-transformed without additional z-score standardization. Bray–Curtis dissimilarity was used for bacterial community composition and predicted functional potential matrices, whereas Euclidean distance was used for environmental variables. Partial least squares path modeling (PLS-PM) was performed using the plspm package to explore association patterns among biochar input, soil chemical attributes, microbial biomass, enzyme activities, bacterial community structure, and predicted functional potential.

## 3 Results

### 3.1 Characteristics of the biochars

SEM images showed that PC had a rough and fractured surface with visible grooves, cracks, and pore-like structures, whereas HC exhibited a fibrous morphology with ridge-like surface textures and irregular particles attached to the surface (Figures 1a,c). The O1s XPS spectra of both PC and HC were deconvoluted into two major components (Figures. 1b,d). The lower-binding-energy component, assigned to C=O/O-C=O/-OH groups, accounted for 64.7% in PC and 67.3% in HC. The higher-binding-energy component, attributed to C-O/C-O-C/O-H and adsorbed oxygen species, accounted for 35.3% in PC and 32.7% in HC.

**Figure 1:**
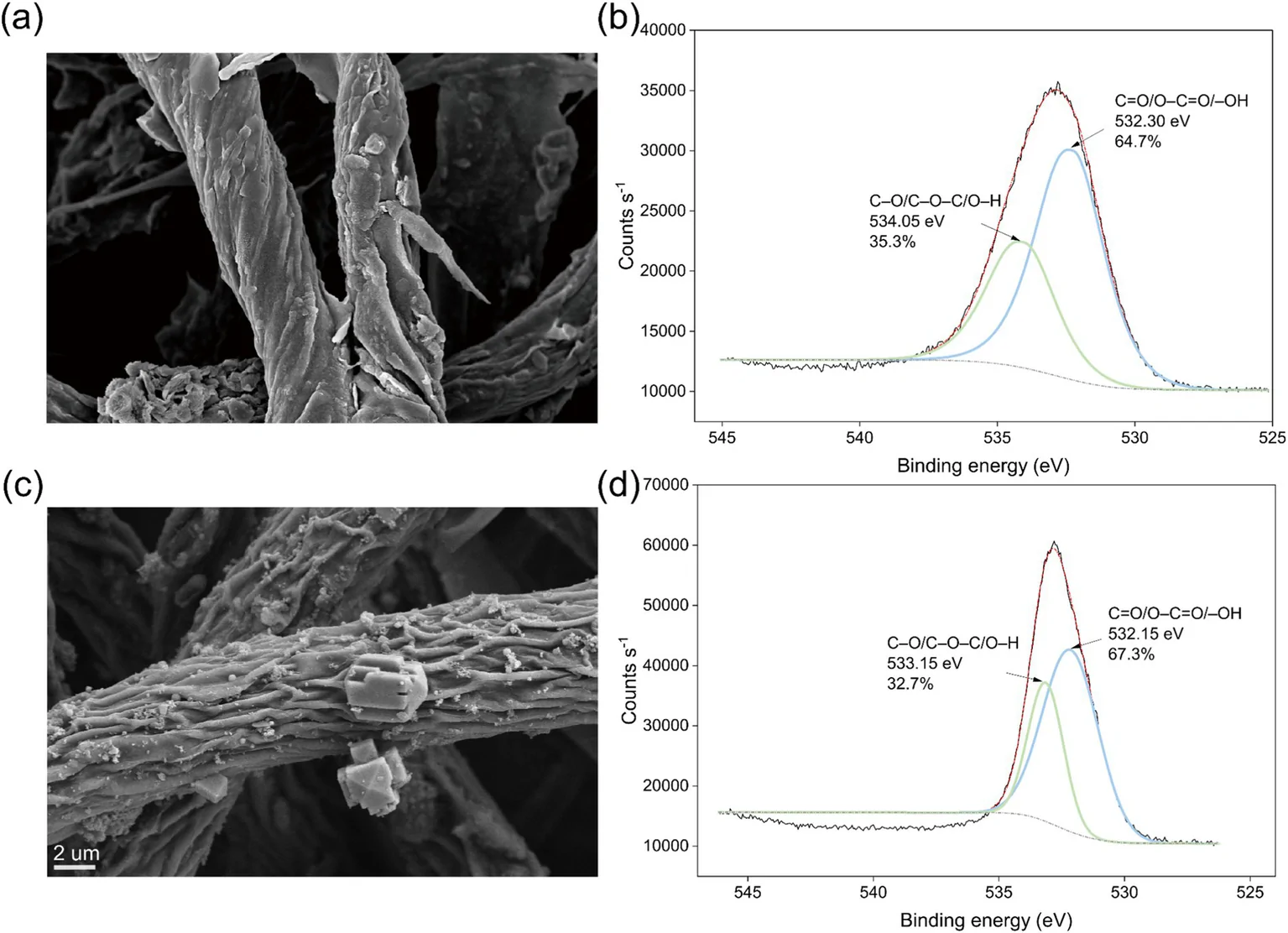
SEM images and O1s XPS spectra of two types of biochar. **(a,b)** pyrochar; **(c,d)** hydrochar.

### 3.2 Effects of PC and HC on soil bacterial community composition under severe NAR stress

As shown in Supplementary Figure S1, a total of 10,975 bacterial ASVs were detected across the 12 soil samples from the *Q. acutissima* plantation, with each ASV showing non-zero abundance in at least one sample. Among them, 190 ASVs were shared among all four treatments, accounting for 1.73% of the total detected ASVs. Among the four treatments, the NAR treatment harbored the highest number of detected ASVs (3,763), followed by HC-NAR and CK with 3,347 and 3,341 ASVs, respectively. In contrast, the PC-NAR treatment showed the lowest number of detected ASVs (2,897). The numbers of treatment-specific ASVs in CK, NAR, PC-NAR, and HC-NAR were 2,330, 2,793, 1,967, and 2,245, respectively, corresponding to 21.23, 25.45, 17.92, and 20.46% of the total detected ASVs.

A total of 35 bacterial phyla were identified across all treatments. Among them, 29 phyla were detected in CK, compared with 28, 26, and 26 phyla in NAR, PC-NAR, and HC-NAR, respectively. Based on relative abundance, nine bacterial phyla were identified as dominant taxa across all treatments, including Acidobacteriota (36.01–38.55%), Proteobacteria (30.87–35.57%), Actinobacteriota (5.52–7.91%), Chloroflexi (3.42–6.37%), Bacteroidota (2.14–3.33%), Gemmatimonadota (2.74–3.03%), Myxococcota (1.98–2.87%), Planctomycetota (2.25–2.66%), and RCP2-54 (1.41–2.15%). The relative abundance of each of these phyla exceeded 1% in all treatments, indicating that they were the dominant bacterial phyla in the soils of the *Q. acutissima* plantation (Figure 2a). Compared with NAR, the relative abundance of Chloroflexi decreased by 41.20 and 38.06% in PC-NAR and HC-NAR, respectively, whereas Actinobacteriota increased by 43.30 and 38.52%, respectively (Figure 2b). In addition, PC-NAR exhibited a higher relative abundance of Acidobacteriota than HC-NAR, whereas Proteobacteria was significantly more abundant in HC-NAR than in NAR and PC-NAR (*p* < 0.05).

**Figure 2:**
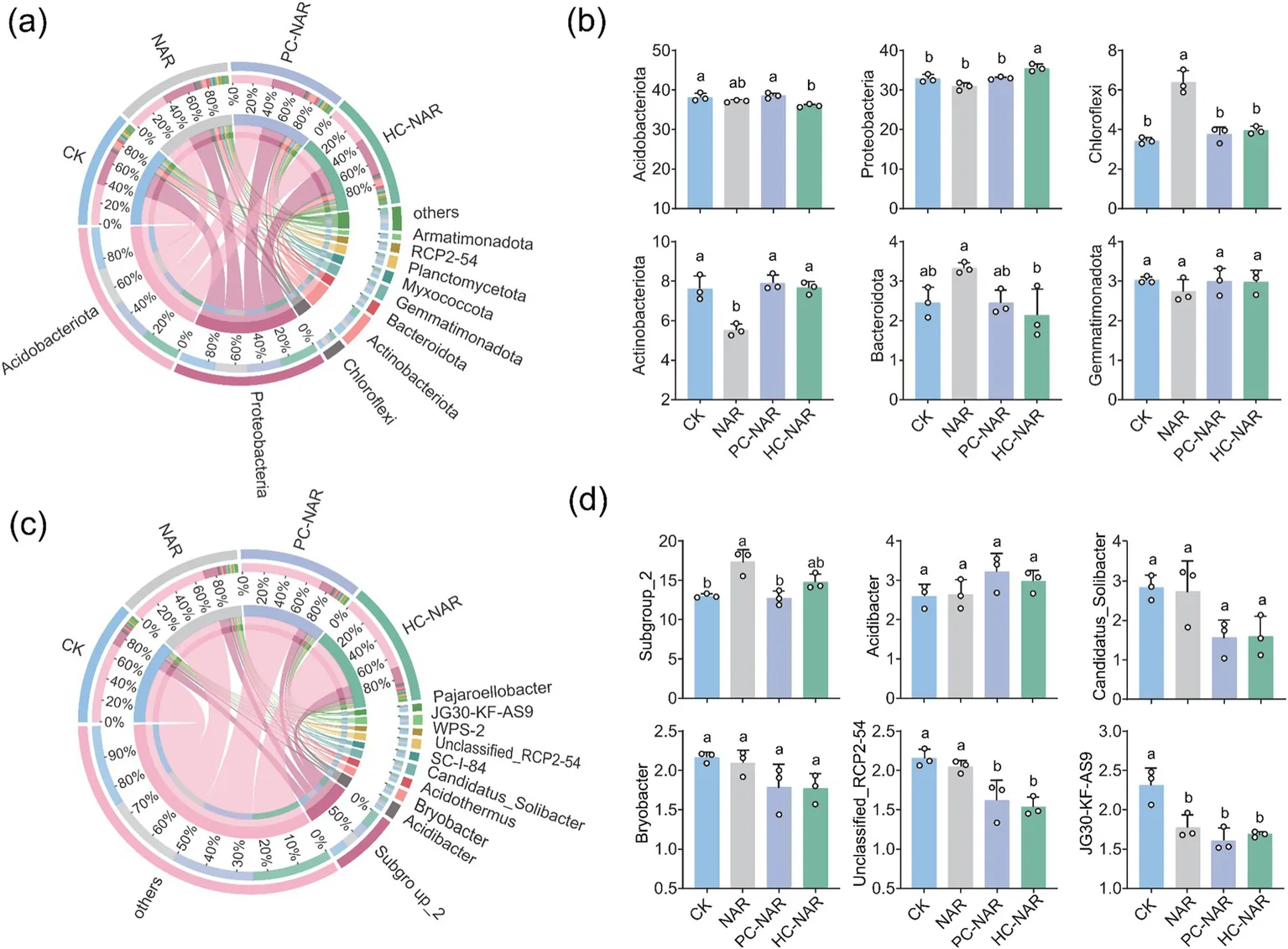
Taxonomic composition of soil bacterial communities at the phylum and genus levels under different treatments. The relative abundances of the top 10 bacterial phyla **(a)** and genus **(c)**. The relative abundances of the top 6 dominant bacterial phyla **(b)** and genus **(d)**. Different lowercase letters indicate significant differences among treatments at *p* < 0.05. CK, control; NAR, nitrate-dominated acid rain; PC-NAR, PC combined with NAR; HC-NAR, HC combined with NAR.

Taxonomic assignment at the genus level revealed 457 bacterial taxa across the soil samples. Subgroup_2 (12.76–17.38%), Acidibacter (2.61–3.21%), Candidatus_Solibacter (1.56–2.83%), Bryobacter (1.77–2.17%), Unclassified_RCP2-54 (1.54–2.15%), JG30-KF-AS9 (1.61–2.31%), and Acidothermus (1.33–1.88%) were the dominant genus-level taxa (Figure 2c). Among them, Subgroup_2 showed the highest relative abundance in the NAR treatment, reaching 17.38%. Compared with NAR, Unclassified_RCP2-54 abundance declined by 20.75% in PC-NAR and by 25.00% in HC-NAR. Relative to CK, the relative abundance of JG30-KF-AS9 was also significantly reduced under PC-NAR and HC-NAR, with decreases of 30.39 and 27.10%, respectively (Figure 2d).

### 3.3 Effects of PC and HC on treatment-associated bacterial taxa under severe NAR stress

LEfSe analysis was performed to identify treatment-associated bacterial taxa based on thresholds of LDA > 4.0 and *p* < 0.05 (Figure 3). The discriminant taxa covered four taxonomic ranks, including three phyla, three classes, three orders, and one family. At the phylum level, Chloroflexi showed higher relative abundance in the NAR treatment, whereas Acidobacteriota and Proteobacteria were preferentially associated with PC-NAR and HC-NAR treatments, respectively. At the class level, Acidobacteriae affiliated with Acidobacteriota, Ktedonobacteria affiliated with Chloroflexi, and Alphaproteobacteria affiliated with Proteobacteria were identified as treatment-associated taxa in CK, NAR, and HC-NAR treatments, respectively. At the order level, Ktedonobacterales affiliated with Chloroflexi, Acidobacteriales affiliated with Acidobacteriota, and Elsterales affiliated with Proteobacteria were enriched in NAR, PC-NAR, and HC-NAR treatments, respectively. Acidobacteriaceae_(Subgroup_1), affiliated with Acidobacteriota, was the only family-level taxon identified by LEfSe and was mainly associated with the CK treatment.

**Figure 3:**
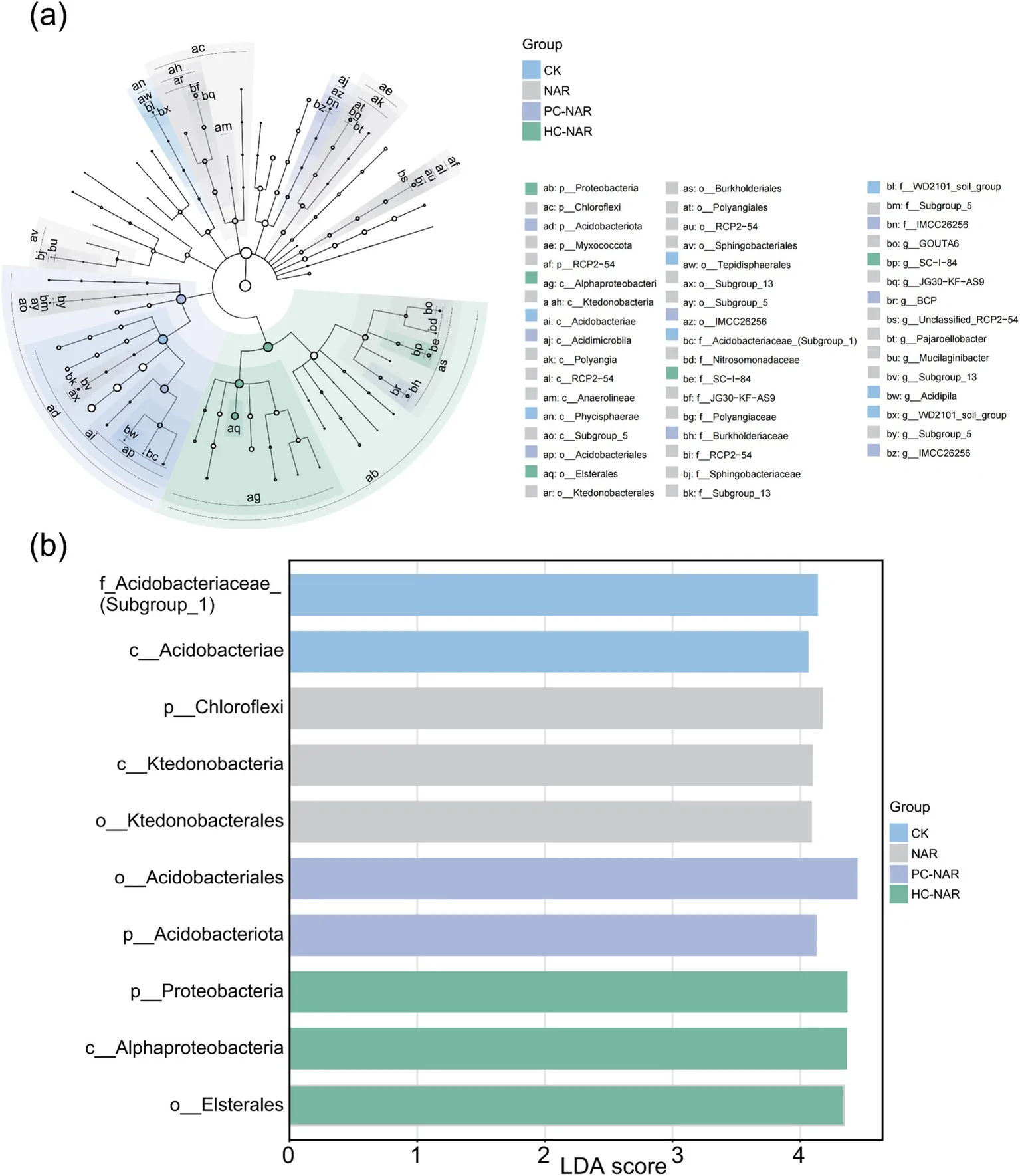
Treatment-associated bacterial taxa identified by LEfSe analysis. **(a)** Cladogram showing differentially abundant bacterial taxa from phylum to genus levels across treatments. **(b)** LDA score plot showing the bacterial taxa associated with each treatment group (LDA > 4.0). CK, control; NAR, nitrate-dominated acid rain; PC-NAR, PC combined with NAR; HC-NAR, HC combined with NAR.

### 3.4 Effects of PC and HC on soil bacterial α- and β-diversity under severe NAR stress

Soil bacterial α-diversity did not differ significantly among treatments (Figure 4a; Supplementary Table S2) (*p* > 0.05), as indicated by the Chao1, ACE, Shannon, and Simpson indices, suggesting that the treatments did not markedly alter bacterial richness or overall diversity. In contrast, PCoA revealed clear treatment-induced differences in bacterial community composition based on Bray–Curtis dissimilarity (Figure 4b). The first and second principal coordinate axes explained 13.94 and 11.78% of the variation in Bray–Curtis dissimilarity, respectively. PERMANOVA revealed a significant effect of treatment on bacterial community composition (pseudo-*F* = 1.427, *R*^2^ = 0.350, *p* = 0.002), while multivariate dispersion did not differ significantly among treatments (PERMDISP: *F* = 2.087, *p* = 0.135). The mean distances to the CK centroid were 0.740, 0.717, and 0.666 for NAR, PC-NAR, and HC-NAR, respectively. Although HC-NAR showed the lowest numerical distance to the CK centroid, the difference among treatments was not statistically significant (*F* = 4.834, *p* = 0.086).

**Figure 4:**
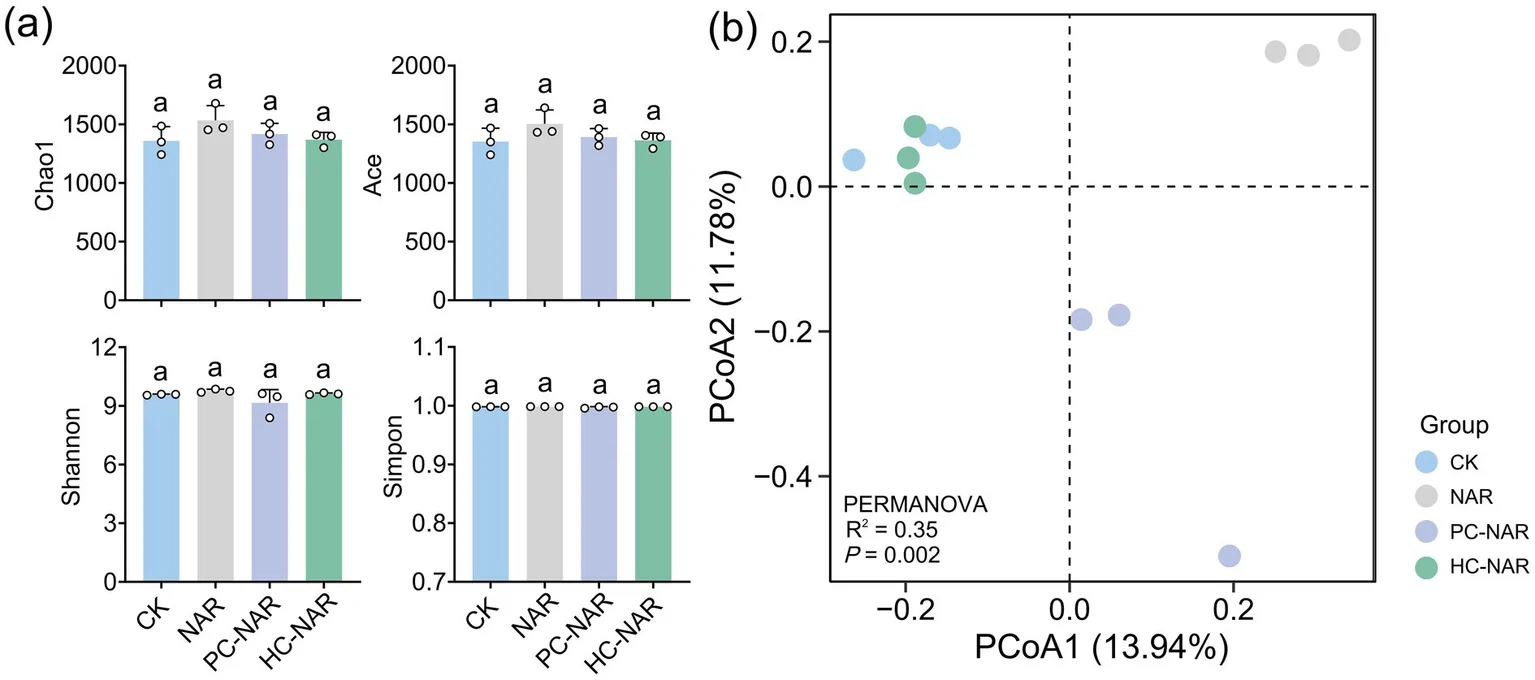
Responses of soil bacterial diversity to different treatments. **(a)** α-Diversity indices. Different lowercase letters indicate significant differences among treatments at *p* < 0.05. **(b)** PCoA ordination based on Bray–Curtis dissimilarity. CK, control; NAR, nitrate-dominated acid rain; PC-NAR, PC combined with NAR; HC-NAR, HC combined with NAR.

### 3.5 Effects of PC and HC on predicted bacterial phenotypes under severe NAR stress

The phenotypic profiles of soil bacterial communities in the *Q. acutissima* plantation were predicted using BugBase (Figure 5). The results showed that Forms_Biofilms was the dominant predicted phenotype across the soil bacterial communities. Relative to NAR, PC-NAR significantly decreased the predicted relative abundances of Contains_Mobile_Elements and Gram_Negative by 5.15 and 1.20%, respectively, but increased those of Gram_Positive and Stress_Tolerant by 21.52 and 14.43%, respectively (*p* < 0.05). HC mainly increased the predicted relative abundance of the facultatively anaerobic phenotype, which was 13.27% higher than that in NAR (*p* < 0.05).

**Figure 5:**
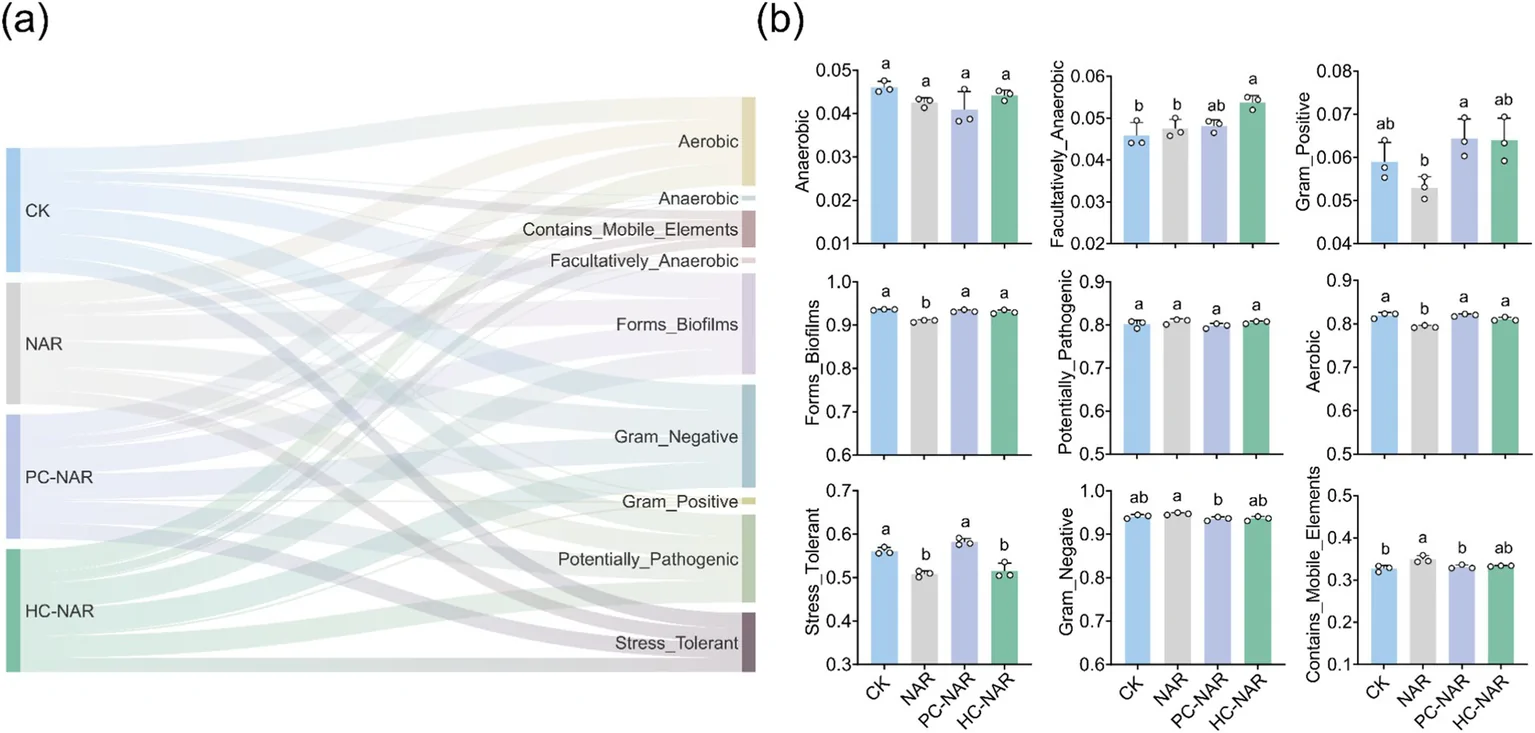
BugBase-predicted phenotypic profiles of soil bacterial communities under different treatments. **(a)** Treatment-specific distribution of predicted bacterial phenotypic traits. **(b)** Relative abundances of predicted bacterial phenotypes. Different lowercase letters indicate significant differences among treatments at *p* < 0.05. CK, control; NAR, nitrate-dominated acid rain; PC-NAR, PC combined with NAR; HC-NAR, HC combined with NAR.

In addition, the NAR treatment significantly suppressed the predicted phenotypic traits Aerobic and Forms_Biofilms compared with all other treatments (*p* < 0.05). Relative to NAR, Aerobic abundance was elevated by 3.30% in CK, 3.38% in PC-NAR, and 2.20% in HC-NAR. The corresponding increases in Forms_Biofilms were 2.88, 2.52, and 2.30%, respectively.

### 3.6 Effects of PC and HC amendments on predicted bacterial functional potential under severe NAR stress

The predicted functional potential of soil bacterial communities was evaluated based on PICRUSt2-predicted KO profiles associated with C, P, N, and S cycling (Figure 6). C cycling contained the largest number of retained KOs, with 69 KOs included in the analysis (Figure 6a). Among the 10 most abundant predicted C-cycling KOs, HC-NAR showed higher predicted abundances of KOs annotated as hexosaminidase and acyl-CoA dehydrogenase by 1.56 and 12.76%, respectively, compared with NAR (*p* < 0.05). P cycling comprised 50 retained KOs (Figure 6b), and HC-NAR exhibited higher predicted abundances of KOs annotated as phoU, ppx, ppnK, ndk, PK, pstB, ugpQ, and relA by 3.54–7.21% relative to NAR (*p* < 0.05). For N cycling, 21 KOs were retained (Figure 6c), and PC-NAR showed higher predicted abundances of KOs annotated as nirD-, nifK-, and nifD-related functions by 3.13, 34.71, and 34.44%, respectively, compared with NAR (*p* < 0.05). S cycling comprised 13 retained KOs (Figure 6d), and both PC-NAR and HC-NAR showed lower predicted abundances of KOs annotated as sat-, dsrA-, aprA-, and dsrB-related functions relative to NAR (*p* < 0.05).

**Figure 6:**
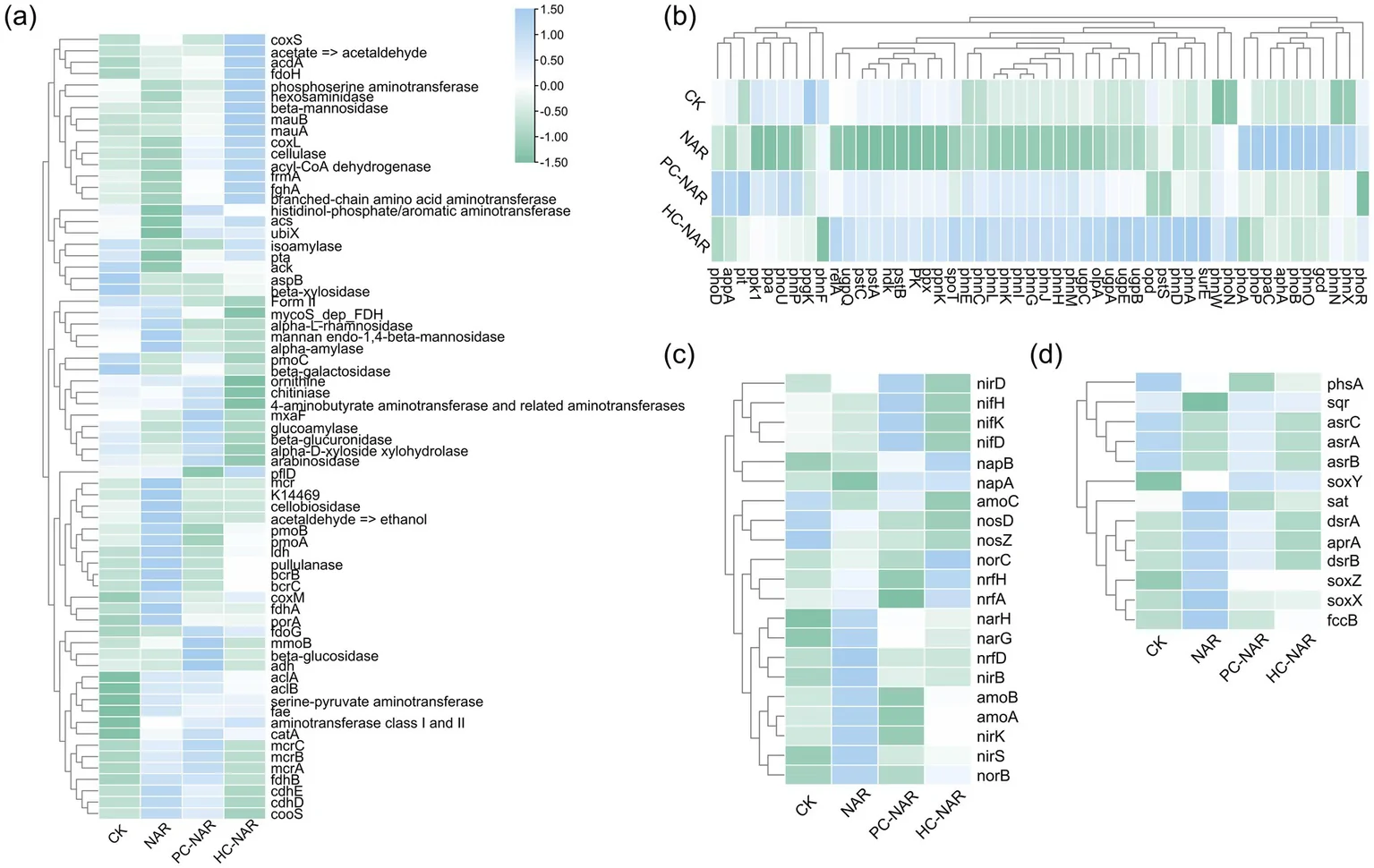
PICRUSt2-predicted KO profiles representing bacterial functional potential associated with carbon **(a)**, phosphorus **(b)**, nitrogen **(c)**, and sulfur **(d)** cycling under different treatments. CK, control; NAR, nitrate-dominated acid rain; PC-NAR, PC combined with NAR; HC-NAR, HC combined with NAR.

### 3.7 Exploratory association patterns linking PC and HC amendments with soil properties and bacterial responses

Mantel tests were used to assess associations between soil environmental factors and bacterial community structure or predicted functional potential (Figure 7). Under PC application, bacterial community structure was significantly associated with pH (*r* = 0.905, *p* = 0.007), SOC (*r* = 0.540, *p* = 0.019), TN (*r* = 0.348, *p* = 0.037), N: P (*r* = 0.370, *p* = 0.033), C: P (*r* = 0.459, *p* = 0.031), and DON (*r* = 0.423, *p* = 0.036). Significant relationships were also found for microbial biomass-related variables, including MBC (*r* = 0.826, *p* = 0.010), MBN (*r* = 0.672, *p* = 0.006), MBN: P (*r* = 0.516, *p* = 0.012), and MBC: P (*r* = 0.680, *p* = 0.008). In contrast, predicted bacterial functional potential was not significantly correlated with any measured environmental variables under PC application (*p* > 0.05).

**Figure 7:**
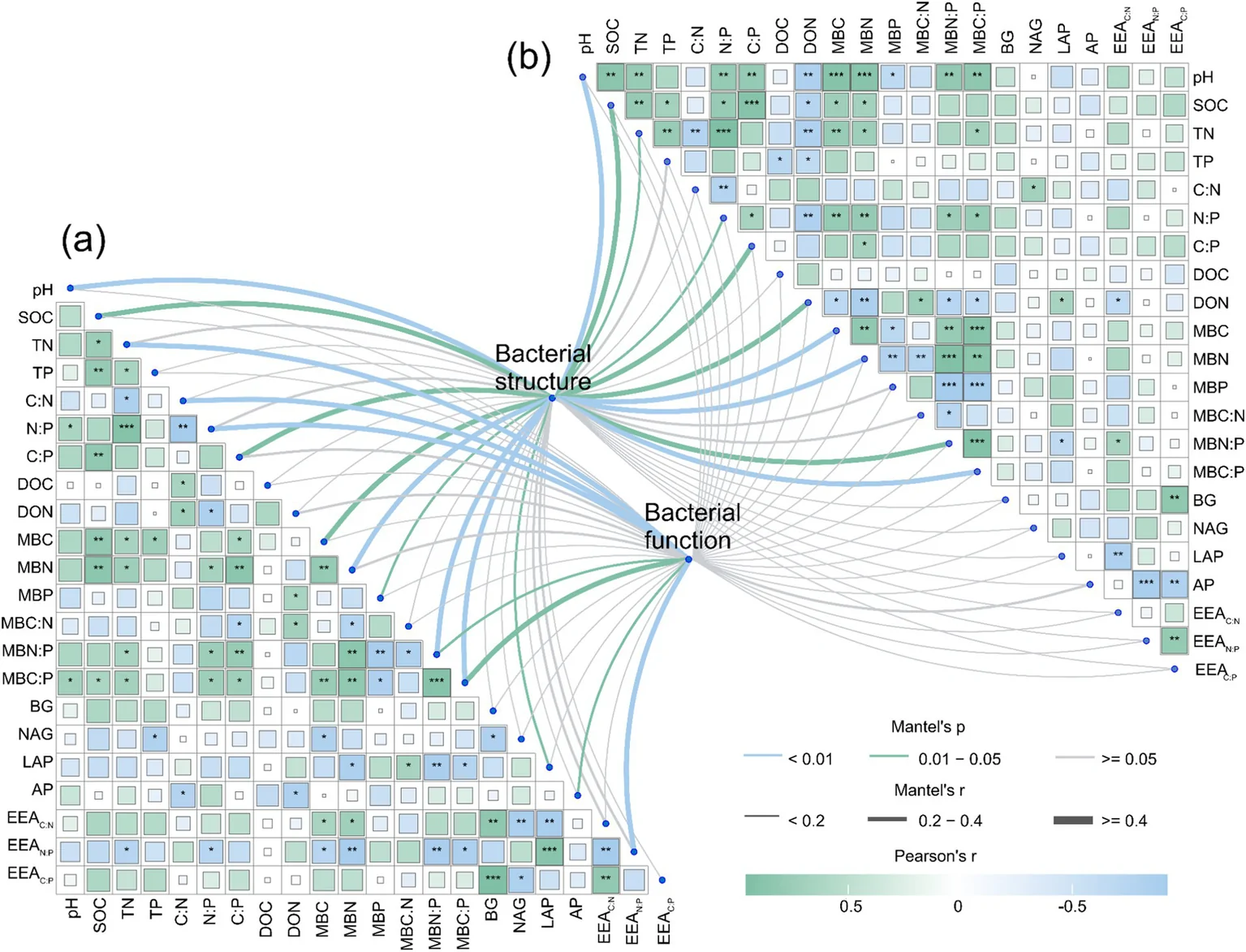
Mantel analysis of the associations between environmental factors and soil bacterial community structure and predicted functional potential under different biochar treatments. *, *p* < 0.05; **, *p* < 0.01; ***, *p* < 0.001. SOC, soil organic carbon; TN, total nitrogen; TP, total phosphorus; C: N, soil organic carbon: total nitrogen ratio; N: P, total nitrogen: total phosphorus ratio; C: P, soil organic carbon: total phosphorus ratio; DOC, dissolved organic carbon; DON, dissolved organic nitrogen; MBC, microbial biomass carbon; MBN, microbial biomass nitrogen; MBP, microbial biomass phosphorus; MBC: N, microbial biomass carbon: microbial biomass nitrogen ratio; MBC: P, microbial biomass carbon: microbial biomass phosphorus ratio; MBN: P, microbial biomass nitrogen: microbial biomass phosphorus ratio; BG, β-glucosidase; NAG, N-acetyl-β-D-glucosaminidase; LAP, leucine aminopeptidase; AP, acid phosphatase; EEA_C: N_, lnBG: ln(NAG+LAP) ratio; EEA_C: P_, lnBG: lnAP ratio; EEA_N: P_, ln(NAG+LAP): lnAP ratio. **(a)** Hydrochar, **(b)** Pyrochar.

Under HC application, bacterial community structure was significantly correlated with pH (*r* = 0.571, *p* = 0.008), SOC (*r* = 0.414, *p* = 0.034), C: P (*r* = 0.498, *p* = 0.020), MBC (*r* = 0.591, *p* = 0.011), MBN (*r* = 0.673, *p* = 0.009), MBP (*r* = 0.302, *p* = 0.048), MBN: P (*r* = 0.726, *p* = 0.003), MBC: P (*r* = 0.788, *p* = 0.001), and LAP activity (*r* = 0.312, *p* = 0.045). Predicted bacterial functional potential was significantly associated with several nutrient stoichiometric and enzymatic variables, including TN (*r* = 0.599, *p* = 0.006), C: N (*r* = 0.597, *p* = 0.003), N: P (*r* = 0.682, *p* = 0.005), MBN: P (*r* = 0.336, *p* = 0.037), MBC: P (*r* = 0.405, *p* = 0.020), AP activity (*r* = 0.358, *p* = 0.035), and EEA_N: P_ (*r* = 0.468, *p* = 0.009).

PLS-PM further indicated distinct association patterns among soil properties, microbial biomass, enzyme activities, bacterial community structure, and predicted functional potential under PC and HC amendments (Figure 8). The PLS-PM models showed exploratory goodness-of-fit values within the present dataset. The PC models for bacterial community structure and predicted functional potential showed goodness-of-fit values of 0.70 and 0.64, respectively. The HC models showed goodness-of-fit values of 0.68 and 0.62, respectively.

**Figure 8:**
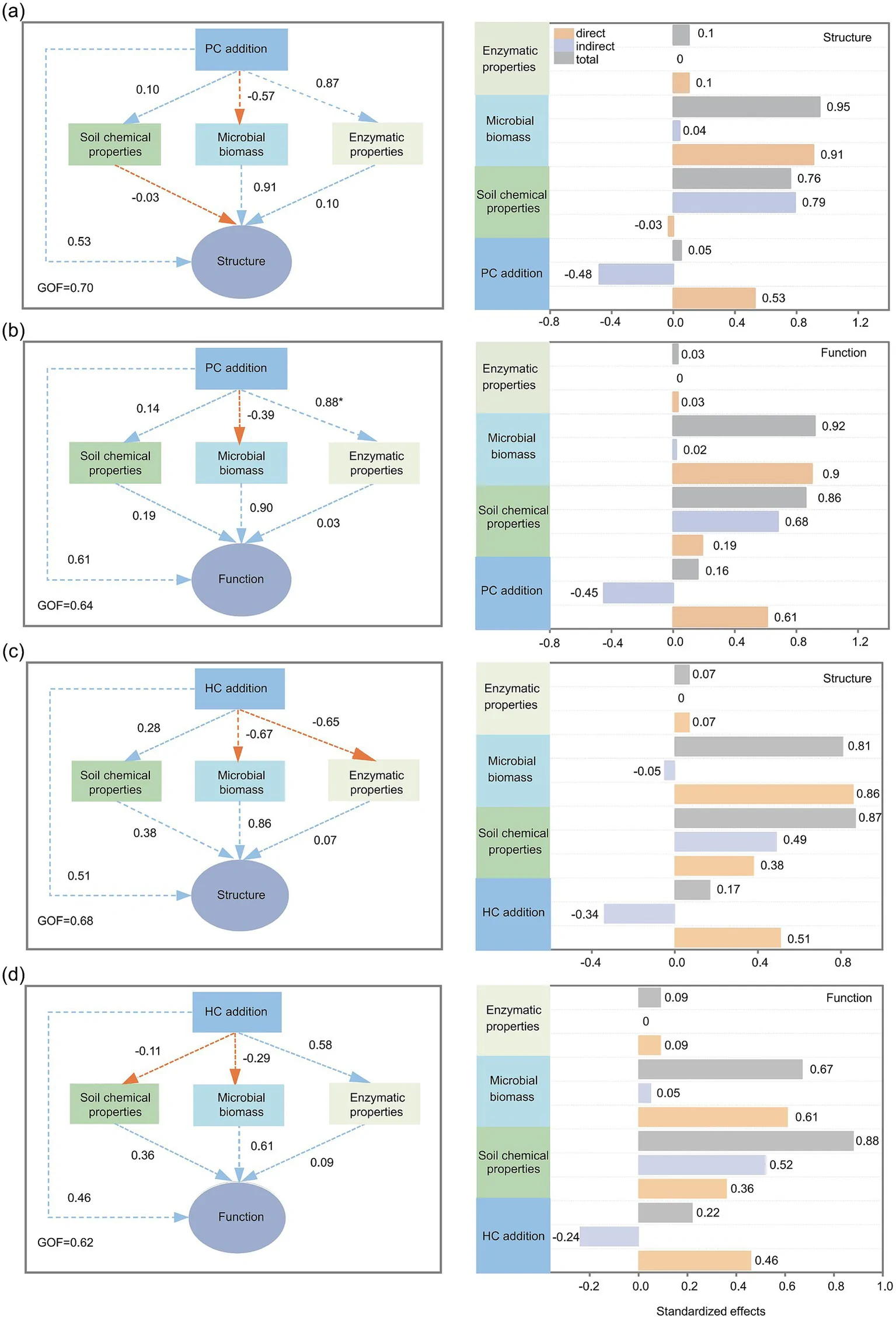
Exploratory partial least squares path models showing association patterns among soil chemical properties, microbial biomass, enzymatic properties, bacterial community structure, and predicted functional potential. The models illustrate the exploratory association patterns under pyrochar addition (PC) and hydrochar addition (HC): **(a)** bacterial community structure under PC addition; **(b)** predicted functional potential under PC addition; **(c)** bacterial community structure under HC addition; and **(d)** predicted functional potential under HC addition. Blue arrows indicate positive associations, and orange arrows indicate negative associations. Numbers on the arrows indicate standardized path coefficients. The bar plots on the right show the standardized direct, indirect, and total effects within each exploratory model. PC, pyrochar; HC, hydrochar.

Under PC amendment, PC addition showed a positive association with bacterial community structure (path coefficient = 0.53). Negative associations were observed between PC addition and microbial biomass in the bacterial community structure and predicted functional potential models, with path coefficients of −0.57 and −0.39, respectively. Enzyme activity showed positive associations in both models, with path coefficients of 0.87 and 0.88, respectively. Among the examined environmental variables, microbial biomass showed relatively stronger associations with bacterial community structure and predicted functional potential under PC amendment, with path coefficients of 0.91 and 0.90, respectively. Standardized total effects further indicated that microbial biomass had relatively higher total effects on bacterial community structure and predicted functional potential, with values of 0.95 and 0.92, respectively.

Under HC amendment, HC showed positive associations with bacterial community structure and predicted functional potential, with path coefficients of 0.51 and 0.46, respectively. HC addition showed a negative association with microbial biomass in the bacterial community structure model (path coefficient = −0.67), whereas the association was weaker in the predicted functional potential model (path coefficient = −0.29). The associations between HC addition and enzyme activity differed between the two models, with path coefficients of −0.65 and 0.58, respectively. Soil chemical properties showed positive associations with bacterial community structure and predicted functional potential, with path coefficients of 0.38 and 0.36, respectively, while microbial biomass showed positive associations with both response variables, with path coefficients of 0.86 and 0.61, respectively. Standardized total effects indicated that soil chemical properties exhibited relatively higher total effects on bacterial community structure and predicted functional potential under HC amendment, with values of 0.87 and 0.88, respectively, followed by microbial biomass, with total effects of 0.81 and 0.67.

## 4 Discussion

### 4.1 Material-dependent biochar traits and bacterial community restructuring under severe NAR stress

The different surface properties of PC and HC may help explain their different effects on bacterial communities under severe NAR stress. SEM observations showed that PC had a rough and fractured surface with visible grooves, cracks, and pore-like structures, whereas HC showed a fibrous morphology with ridge-like surface textures and irregular particles attached to the surface (Figures 1a,c). The O1s XPS spectra showed that both biochars contained oxygen-containing surface groups, and HC had a slightly higher proportion of the lower-binding-energy component assigned to C=O/O-C=O/-OH groups than PC (Figures 1b,d). These differences suggest that PC and HC may provide different surface conditions for microbial colonization and soil-biochar interactions, which may contribute to the different bacterial community responses observed under PC-NAR and HC-NAR treatments.

Consistent with these material differences, our results showed that severe NAR stress reshaped the soil bacterial community in *Q. acutissima* plantations, while PC-NAR and HC-NAR exhibited distinct bacterial community compositions relative to the NAR treatment. Compared with CK, the NAR treatment increased the total number of bacterial ASVs and treatment-specific ASVs (Supplementary Figure S1). This response may indicate stress-induced community differentiation under severe NAR, probably due to stronger environmental filtering associated with soil acidification (Zhou et al., 2022). However, Acidobacteriota and Proteobacteria were consistently identified as the dominant bacterial phyla across treatments, indicating that these major phyla maintained similar patterns of relative dominance under different NAR and biochar amendment conditions. Acidobacteriota are commonly adapted to acidic and oligotrophic conditions, while Proteobacteria often respond rapidly to changes in substrate availability and environmental conditions (Kang et al., 2022; Xu et al., 2025). It should be noted that the simulated NAR treatment simultaneously introduced proton acidity and reactive N and S inputs. Therefore, the individual contributions of acidity, N deposition, and S deposition could not be disentangled within the present experimental design. The observed bacterial responses should therefore be interpreted as the integrated effects of severe nitrate-dominated acid deposition rather than the independent effects of individual chemical components.

Compared with NAR, both PC-NAR and HC-NAR reduced the relative abundance of Chloroflexi and increased the relative abundance of Actinobacteriota (Figure 2). LEfSe analysis further identified Chloroflexi-related taxa as treatment-associated taxa of NAR stress, whereas Acidobacteriota- and Proteobacteria-related taxa were enriched under PC-NAR and HC-NAR, respectively (Figure 3). These shifts indicate that PC and HC altered bacterial community assembly under severe NAR stress. The enrichment of different indicator taxa under PC-NAR and HC-NAR also suggests that the two biochar types imposed different ecological filters. These differences may be linked to their contrasting material properties, including aromaticity, pore structure, surface functionality, and labile organic components (Supplementary Table S1). Similar biochar-induced bacterial shifts have been reported in previous studies, although the direction and magnitude of these responses can vary with feedstock, carbonization conditions, application rate, soil type, and background environmental conditions (Shu et al., 2023; Kracmarova-Farren et al., 2024; Cao et al., 2025).

Although the overall treatment effect on bacterial community composition was significant, pairwise PERMANOVA comparisons did not reveal significant differences among individual treatment pairs, and multivariate dispersion was comparable across treatments. HC-NAR exhibited a numerically lower distance to the CK centroid than NAR and PC-NAR, although the difference was not statistically significant (Figure 4b). Therefore, HC-NAR showed a tendency toward a bacterial community composition more similar to CK. The observed similarity may reflect differences in microbial responses to HC amendment, potentially related to changes in soil physicochemical conditions and resource availability (Yan et al., 2024). In contrast, PC may reshape bacterial communities mainly by enhancing adsorption-related processes and creating stable microsites for microbial colonization (Bolan et al., 2024; Zhao et al., 2024). These material-dependent effects may help explain the different bacterial community trajectories observed under PC and HC amendments. In contrast to *β*-diversity, *α*-diversity indices did not differ significantly among treatments (Figure 4a). Therefore, the bacterial response to PC and HC was mainly reflected in community restructuring rather than in changes in richness or overall diversity. The limited *α*-diversity response may be related to the relatively short experimental duration, and longer-term field studies are needed to verify the persistence of these biochar-mediated effects (Li et al., 2026).

### 4.2 Predicted phenotypic and functional responses to PC and HC amendments

The predicted phenotypic profiles further characterized potential bacterial community responses to severe NAR stress and biochar amendment (Figure 5). Forms_Biofilms was the predominant predicted phenotype across the forest soil bacterial communities, indicating a relatively high predicted taxonomic potential for biofilm-associated traits. Under NAR, the predicted relative abundances of Aerobic and Forms_Biofilms were lower than those under the other treatments. These patterns suggest shifts in the predicted representation of bacterial taxa associated with these phenotypic categories under severe NAR stress. However, because these phenotypes were inferred using BugBase from 16S rRNA gene data rather than directly measured, their ecological significance should be interpreted cautiously and requires physiological validation (Deng et al., 2022; Wu et al., 2019; Shi et al., 2023).

PC and HC produced different predicted phenotypic responses. PC increased the predicted relative abundances of Gram_Positive and Stress_Tolerant phenotypes, while decreasing those of Gram_Negative and Contains_Mobile_Elements (Figure 5). This shift suggests that PC may alleviate severe NAR stress by modifying environmental filtering and favoring bacterial groups with higher stress tolerance (Xia et al., 2023). Such a response may be related to the relatively stable structure and microsite-forming capacity of PC. HC mainly increased the predicted relative abundance of Facultatively_Anaerobic bacteria. This response suggests that HC may change bacterial ecological strategies by altering substrate availability and microscale oxygen conditions. The relatively labile organic components of HC may provide more accessible carbon sources and increase microenvironmental heterogeneity, thereby favoring bacteria that can adapt to variable oxygen conditions (Yan et al., 2024). However, these interpretations should be treated with caution because BugBase provides predicted phenotypic profiles rather than direct physiological measurements. Future studies should validate these patterns using cultivation-based assays, physiological measurements, and multi-omics approaches.

The predicted functional profiles revealed material-dependent differences in bacterial functional potential between PC and HC treatments (Figure 6). HC showed higher predicted abundances of several KOs associated with carbon metabolism, suggesting that HC-NAR and PC-NAR treatments exhibited different patterns of predicted carbon-cycling functional potential (Ji et al., 2020). In contrast, PC-NAR exhibited higher predicted abundances of KOs annotated with nirD-, nifK-, and nifD-related functions, which are involved in nitrogen transformation and fixation-related associations (Jia et al., 2026). These patterns indicate differences in predicted N-cycling functional potential under PC-NAR but do not provide direct evidence for enhanced nitrogen fixation or nitrogen transformation rates (Feng et al., 2026). For P cycling, HC-NAR showed higher predicted abundances of KOs associated with phosphate transport, polyphosphate metabolism, and phosphorus homeostasis, indicating differences in predicted phosphorus-related functional potential. The distinct chemical properties of HC, including its nutrient composition and organic functional characteristics, may contribute to these predicted functional patterns by altering soil resource availability and microbial habitat conditions (Gong et al., 2023).

Both PC-NAR and HC-NAR showed lower predicted abundances of several S-cycling KOs compared with NAR. These differences should be interpreted as shifts in predicted S-cycling functional potential rather than direct changes in sulfur transformation processes. Variations in soil acidification status, redox conditions, and substrate availability may contribute to these predicted patterns (Gong et al., 2023; Zhang et al., 2025). Importantly, PICRUSt2 infers gene-family functional potential from 16S rRNA gene profiles and does not directly measure functional gene abundance, gene expression, nitrogen fixation, or biogeochemical process rates. Therefore, the predicted functional differences identified here require further validation using metagenomics, metatranscriptomics, targeted functional-gene quantification, or process-based measurements.

### 4.3 Distinct association patterns of PC and HC under NAR stress

The Mantel tests and PLS-PM analyses revealed distinct association patterns among soil chemical properties, microbial biomass, bacterial community composition, and predicted functional potential under PC and HC amendments (Figures 7, 8). Under PC amendment, microbial biomass variables showed relatively stronger associations with bacterial community composition and predicted functional potential than most individual soil chemical variables. These relationships suggest that microbial biomass was closely associated with PC-related bacterial responses under NAR stress.

The distinct association pattern under PC amendment may be related to the physicochemical characteristics of pyrochar. Previous studies have reported that pyrochar generally possesses higher aromaticity, developed pore structures, and strong adsorption capacity (Xia et al., 2023). These characteristics have been suggested to influence microbial habitats and resource availability in soil systems (Yang et al., 2022; Zhu et al., 2026). Therefore, the observed association between microbial biomass and bacterial responses under PC amendment may partly reflect differences in the soil environment associated with PC addition. However, these underlying processes were not directly measured in the present study and require further validation.

A different as sociation pattern was observed under HC amendment. Bacterial community composition showed relatively stronger associations with several soil chemical properties, whereas predicted functional potential was associated with C, N, and selected enzyme activity indices (Figure 7). In addition, PLS-PM provided exploratory insights into the relationships among soil chemical properties, microbial biomass, and bacterial responses under HC amendment (Figure 8). These results suggest that HC-associated bacterial responses were more closely related to variations in soil chemical conditions than those observed under PC amendment. The distinct physicochemical characteristics of hydrochar, including the relatively high abundance of oxygen-containing functional groups and labile organic components reported in previous studies, may provide a possible explanation for these association patterns (Lang et al., 2024; Sun et al., 2020; Ighalo et al., 2025). Such properties have been reported to influence soil nutrient conditions and organic substrate availability, which may subsequently be associated with microbial community responses (Deshoux et al., 2023; Pan et al., 2026; Liu et al., 2026). Nevertheless, the Mantel and PLS-PM results represent statistical associations rather than causal relationships, and the proposed mechanisms require further validation through direct measurements of substrate availability, microbial activity, and related biogeochemical processes.

Overall, PC- and HC-amended soils exhibited contrasting association patterns under severe NAR stress: PC-associated bacterial responses were more closely related to microbial biomass, whereas HC-associated responses showed stronger relationships with soil chemical conditions and their interactions with microbial biomass. These exploratory association patterns provide preliminary insights into the potential mechanisms underlying the contrasting responses of bacterial communities to PC and HC amendments (Figure 9), while the underlying mechanisms remain to be further experimentally validated.

**Figure 9:**
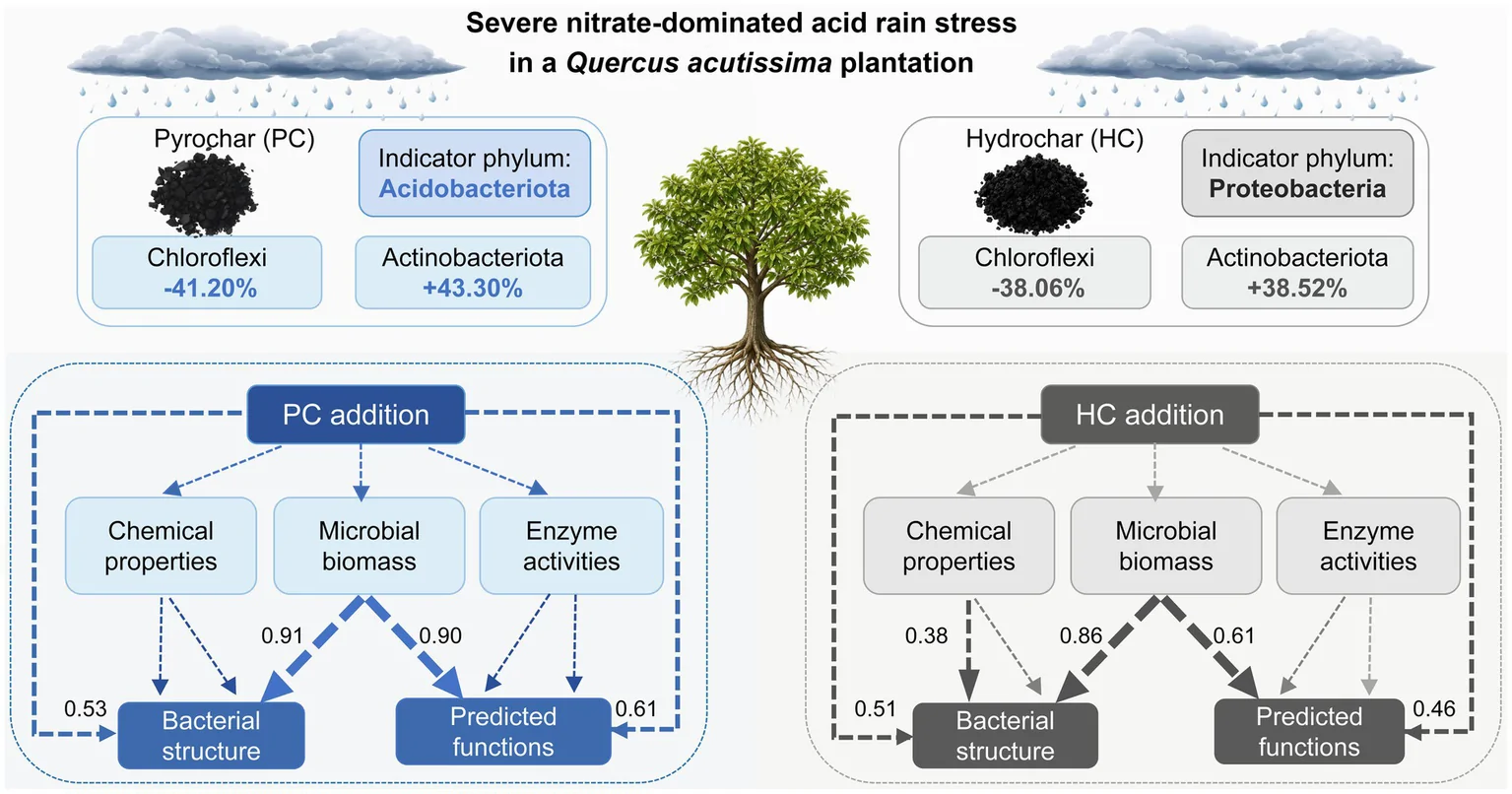
The conceptual diagram of soil bacterial community responses to pyrochar and hydrochar addition in *Quercus acutissima* plantations under severe nitrate-dominated acid rain stress.

### 4.4 Research limitations and implications

Although this study provides evidence that PC and HC differentially influence soil bacterial communities and predicted functional potential under severe NAR stress, several limitations should be considered. First, the BugBase- and PICRUSt2-derived results were inferred from 16S rRNA gene data and therefore represent predicted functional potential rather than direct measurements of microbial metabolism or soil biogeochemical processes. Moreover, the absence of archived information on the standalone PICRUSt2 version, reference database release, and sample-level NSTI values precluded a quantitative assessment of phylogenetic prediction reliability. This uncertainty is particularly relevant for forest-soil bacterial taxa that may be phylogenetically distant from available reference genomes. In addition, multiple-testing correction was not applied in the LEfSe analysis; therefore, the identified treatment-associated taxa should be interpreted as exploratory treatment-associated taxa rather than definitive indicators. Future studies integrating amplicon-based predictions with metagenomics, targeted functional gene quantification, enzyme assays, and process-rate measurements are therefore needed to validate the inferred microbial functions involved in C, N, P, and S cycling.

Second, this short-term field simulation mainly reflects the early responses of NAR-stressed plantation soils to PC and HC amendments. Because biochar aging may alter surface functional groups, adsorption capacity, nutrient release, and microbial habitat properties, long-term field observations are required to determine whether the contrasting responses associated with PC and HC persist under continuous acid deposition. In addition, the absence of PC-only and HC-only treatments prevents separation of the intrinsic effects of each amendment from its interaction with severe NAR stress. Therefore, the responses observed in this study should be interpreted specifically as amendment-associated effects under severe NAR conditions rather than as general effects of PC or HC independent of acid deposition. Future factorial experiments, including amendment-only, NAR-only, and amendment with NAR treatments, would help distinguish their direct and interactive effects. Despite these limitations, this study suggests that PC and HC are not functionally equivalent amendments, but may alleviate NAR-induced soil stress through distinct associations with soil chemical properties and microbial responses, providing useful implications for the targeted application of biochar in NAR-affected plantation soils.

## 5 Conclusion

This study suggested that severe nitrate-dominated acid rain (NAR) was associated with changes in soil bacterial community composition and predicted functional potential in a *Quercus acutissima* plantation. Pyrochar (PC) and hydrochar (HC) exhibited distinct associations with bacterial community responses under NAR conditions. Exploratory PLS-PM analysis suggested that PC-associated bacterial responses were more closely linked to microbial biomass, whereas HC-associated responses showed stronger associations with soil chemical conditions and their interactions with microbial biomass. These findings provide preliminary evidence that PC and HC may induce different microbial response patterns under NAR stress. Furthermore, further validation using larger-scale experiments and direct functional measurements is required.

## Data Availability

The datasets presented in this study can be found in online repositories. The names of the repository/repositories and accession number(s) can be found in the article/Supplementary material.
