# Supplementary material for: Pyrochar versus hydrochar: contrasting modulation of soil bacterial communities under severe nitrate-dominated acid rain stress in a Quercus acutissima plantation
Source: Front Microbiol. 2026 Sep 29;17:1961661. doi: 10.3389/fmicb.2026.1961661 (PMC13658203; doi:10.3389/fmicb.2026.1961661)
Supplement: Supplementary file 1 [file Table_1.DOCX]

**Pyrochar versus hydrochar: Contrasting modulation of soil bacterial communities under severe nitrate-dominated acid rain stress in a *Quercus acutissima* plantation**

Yuanyuan Feng ^a, b, #^, Shushu Yao^a, #^, Ke Yuan^a^, Haibo Hu^a, *^, Peng Cui^a, c, *^, Yanfang Feng^b^, Lihong Xue^b^

**a** Co-Innovation Center for Sustainable Forestry in Southern China, College of Forestry and Grassland, Nanjing Forestry University, Nanjing 210037, China

**b** Key Laboratory of Agro-Environment in Downstream of Yangtze Plain, National Agricultural Experiment Station for Agricultural Environment (Luhe), Ministry of Agriculture and Rural Affairs; Institute of Agricultural Resources and Environment, Jiangsu Academy of Agricultural Sciences, Nanjing 210014, China

**c** Department of Applied Physics and Electronics, Umea University, Umea 90187, Sweden

^#^ These authors contributed equally to this paper.

^*^Corresponding author. Tel.: +86-25-84391526;

E-mail: huhaibo@njfu.edu.cn; cui.peng@umu.se

Supplementary material for Text.

**Soil sampling and determination of soil chemical and biological properties**

Soil samples were collected on 30 June 2022, approximately two weeks after the final simulated NAR application in mid-June 2022, following one year of simulated NAR treatment. Surface litter was removed before sampling. In each plot, five soil cores were collected from the four corners and the center at a depth of 0-10 cm and combined into one composite sample. Each composite sample represented one independent experimental plot, resulting in 12 independent composite samples (three independent field replicates per treatment). After transport to the laboratory, visible stones, roots, plant residues, and soil fauna remains were removed, and each sample was homogenized and divided into three subsamples.

One fresh subsample was transferred to sterile centrifuge tubes and stored at -80 °C for DNA extraction and bacterial community sequencing. A second fresh subsample was temporarily stored at 4 °C for analyses of dissolved organic carbon (DOC), dissolved organic nitrogen (DON), microbial biomass, and enzyme activities. DOC and DON were determined after extraction. The activities of β-glucosidase (BG), β-N-acetylglucosaminidase (NAG), and acid phosphatase (AP) were determined using a p-nitrophenyl colorimetric method, whereas leucine aminopeptidase (LAP) activity was measured using a fluorogenic substrate method. Microbial biomass carbon, nitrogen, and phosphorus (MBC, MBN, and MBP, respectively) were determined using a modified chloroform fumigation-K_2_SO_4_ extraction method. The remaining subsample was air-dried, ground, and sieved for soil physicochemical analyses.

Soil pH was measured using a glass-electrode pH meter at a soil-to-water ratio of 1:2.5. Soil organic carbon (SOC) was determined by potassium dichromate oxidation with external heating. Total nitrogen (TN) was measured using an automatic Kjeldahl analyzer, and total phosphorus (TP) was determined by alkali fusion followed by molybdenum–antimony colorimetric spectrophotometry.

Supplementary material for Table.

**Table S1** Physicochemical characteristics of Pyrochar and Hydrochar.

| **Parameter** | **Pyrochar** | **Hydrochar** |
| --- | --- | --- |
| Organic carbon (g kg⁻^1^) | 589.8 | 450.6 |
| Total nitrogen (g kg⁻^1^) | 4.6 | 5.3 |
| C/N ratio | 128.2 | 85.0 |
| Total phosphorus (g kg⁻^1^) | 0.8 | 1.1 |
| K (g kg⁻^1^) | 5.7 | 6.8 |
| Ca (g kg⁻^1^) | 8.4 | 6.9 |
| Fe (mg kg⁻^1^) | 230.1 | 312.3 |
| Mn (mg kg⁻^1^) | 193.0 | 219.2 |
| pH | 10.0 | 5.6 |
| O/C | 0.08 | 0.32 |
| H/C | 0.42 | 0.88 |
| Ash (%) | 11.2 | 16.5 |
| C (atomic %) | 78.13 | 77.32 |
| O (atomic %) | 18.79 | 20.16 |
| N (atomic %) | 2.77 | 2.36 |
| P (atomic %) | 0.30 | 0.17 |
| Pore diameter (nm) | 1.36 | 1.34 |
| Pore volume (cm^3^ g⁻^1^) | 0.008 | 0.01 |
| Specific surface area (m^2^ g⁻^1^) | 1.80 | 3.26 |

**Table S2** Monthly simulated NAR application volumes and corresponding N, S, and H⁺ inputs during the experimental period.

| **Month** | **Spray volume (L plot⁻^1^)** | **N input (g plot⁻^1^)** | **S input (g plot⁻^1^)** | **H^+^ input (mol plot⁻^1^)** |
| --- | --- | --- | --- | --- |
| Jan | 26.82 | 0.42 | 0.19 | 0.08 |
| Feb | 29.79 | 0.47 | 0.22 | 0.09 |
| Mar | 41.16 | 0.65 | 0.30 | 0.13 |
| Apr | 41.88 | 0.66 | 0.30 | 0.13 |
| May | 47.04 | 0.74 | 0.34 | 0.15 |
| Jun | 99.59 | 1.57 | 0.72 | 0.31 |
| Jul | 106.20 | 1.67 | 0.76 | 0.34 |
| Aug | 82.80 | 1.30 | 0.60 | 0.26 |
| Sep | 39.77 | 0.63 | 0.29 | 0.13 |
| Oct | 29.76 | 0.47 | 0.22 | 0.09 |
| Nov | 28.86 | 0.45 | 0.21 | 0.09 |
| Dec | 17.89 | 0.28 | 0.13 | 0.06 |
| **Annual total** | **591.56** | **9.31** | **4.26** | **1.87** |

**Table S3** Results of Levene’s test for homogeneity of variances of dominant bacterial taxa before linear mixed model analysis.

| **Taxonomic level** | **Taxon** | **Levene statistic** |  | **P value** |
| --- | --- | --- | --- | --- |
| Phylum | Acidobacteriota | 0.98 |  | 0.46 |
| Phylum | Proteobacteria | 0.06 |  | 0.98 |
| Phylum | Actinobacteriota | 0.90 |  | 0.48 |
| Phylum | Chloroflexi | 0.37 |  | 0.78 |
| Phylum | Gemmatimonadota | 0.05 |  | 0.99 |
| Phylum | Bacteroidota | 0.83 |  | 0.52 |
| Genus | Subgroup_2 | 0.33 |  | 0.80 |
| Genus | Acidibacter | 0.122 |  | 0.95 |
| Genus | Candidatus_Solibacter | 0.17 |  | 0.91 |
| Genus | Bryobacter | 0.48 |  | 0.71 |
| Genus | RCP2-54 | 0.30 |  | 0.83 |
| Genus | JG30-KF-AS9 | 0.37 |  | 0.78 |

**Table S4** Alpha diversity indices of soil bacterial communities under different treatments.

| **Index** | **CK** | **NAR** | **PC-NAR** | **HC-NAR** |
| --- | --- | --- | --- | --- |
| Chao1 | 1359.38±70.13a | 1533.98±72.33a | 1417.58±52.09a | 1369.97±34.93a |
| Ace | 1352.49±65.86a | 1504.16±68.41a | 1391.12±41.62a | 1363.49±35.16a |
| Shannon | 9.58±0.02a | 9.77±0.05a | 9.16±0.39a | 9.62±0.03a |
| Simpson | 0.998±0.00a | 0.998±0.00a | 0.997±0.00a | 0.998±0.00a |
| Goods_coverage | 0.995±0.00a | 0.994±0.00a | 0.994±0.00a | 0.995±0.00a |
| Observed_features | 1283.33±45.18b | 1422.00±55.56a | 1319.5±29.16ab | 1308±30.51ab |

Values are means ± standard errors. Different lowercase letters indicate significant differences among treatments after Tukey adjustment based on block-adjusted models (*P* < 0.05). CK, control; NAR, nitrate-dominated acid rain; PC-NAR, PC combined with NAR; HC-NAR, HC combined with NAR.

**Table S5** Rarefied sequencing depth and Good’s coverage of bacterial 16S rRNA gene amplicon sequences for individual soil samples.

| **Sample** | **Treatment** | **Rarefied sequencing depth** | **Good’s coverage (%)** |
| --- | --- | --- | --- |
| CK1 | CK | 24,141 | 99.45 |
| CK2 | CK | 24,141 | 99.30 |
| CK3 | CK | 24,141 | 99.69 |
| NAR1 | NAR | 24,141 | 99.44 |
| NAR2 | NAR | 24,141 | 99.44 |
| NAR3 | NAR | 24,141 | 99.22 |
| PC-NAR1 | PC-NAR | 24,141 | 100.00 |
| PC-NAR2 | PC-NAR | 24,141 | 99.32 |
| PC-NAR3 | PC-NAR | 24,141 | 99.55 |
| HC-NAR1 | HC-NAR | 24,141 | 99.47 |
| HC-NAR2 | HC-NAR | 24,141 | 99.53 |
| HC-NAR3 | HC-NAR | 24,141 | 99.59 |

CK, control; NAR, nitrate-dominated acid rain; PC-NAR, PC combined with NAR; HC-NAR, HC combined with NAR.

**Table S6** KEGG Orthologs (KOs) retained for predicted carbon, nitrogen, phosphorus, and sulfur cycling analyses.

| **Element cycle** | **KO ID** | **Gene name** | **Putative function** | **Functional process** |
| --- | --- | --- | --- | --- |
| Carbon | K00194 | cdhD | Wood Ljungdahl pathway | Carbon fixation |
| Carbon | K00197 | cdhE | Wood Ljungdahl pathway | Carbon fixation |
| Carbon | K00198 | cooS | Wood Ljungdahl pathway | Carbon fixation |
| Carbon | K05825 | aminotransferase class I and II | Aminotransferase class I and II | Organic carbon oxidation |
| Carbon | K00830 | serine-pyruvate aminotransferase/archaeal aspartate aminotransferase | Serine-pyruvate aminotransferase/archaeal aspartate aminotransferase | Organic carbon oxidation |
| Carbon | K00831 | phosphoserine aminotransferase | Phosphoserine aminotransferase | Organic carbon oxidation |
| Carbon | K00812 | aspB | Metabolism of organic sulfur \| Cysteine <-> 3-mercaptopyruvate | Organic carbon oxidation |
| Carbon | K00817 | histidinol-phosphate/aromatic aminotransferase | Histidinol-phosphate/aromatic aminotransferase | Organic carbon oxidation |
| Carbon | K00819 | ornithine/acetylornithine aminotransferase | Ornithine/acetylornithine aminotransferase | Organic carbon oxidation |
| Carbon | K00656 | pflD | Pyruvate <=> acetyl-CoA + formate | Fermentation |
| Carbon | K05989 | alpha-L-rhamnosidase | Hemicullulose debranching | Organic carbon oxidation |
| Carbon | K01183 | chitiniase | Chitin degrading | Organic carbon oxidation |
| Carbon | K01188 | beta-glucosidase | Cellulose degrading | Organic carbon oxidation |
| Carbon | K15230 | aclA | Reverse TCA cycle | Carbon fixation |
| Carbon | K15231 | aclB | Reverse TCA cycle | Carbon fixation |
| Carbon | K04113 | bcrB | Benzoyl-CoA reduction | Organic carbon oxidation |
| Carbon | K04112 | bcrC | Benzoyl-CoA reduction | Organic carbon oxidation |
| Carbon | K19668 | cellobiosidase | Cellulose degrading | Organic carbon oxidation |
| Carbon | K00401 | mcrB | Methane production | Methanogenesis |
| Carbon | K03520 | coxL | Aerobic CO oxidation | Organic carbon oxidation |
| Carbon | K01905 | acdA | Acetogenesis | Fermentation |
| Carbon | K10713 | fae | Formaldehyde oxidation | Organic carbon oxidation |
| Carbon | K01070 | fghA | Formaldehyde oxidation | Organic carbon oxidation |
| Carbon | K00016 | ldh | Lactate utilization | Fermentation |
| Carbon | K10946 | pmoC | Methane oxidation - Partculate methane monooxygenase | Methanotrophy |
| Carbon | K10945 | pmoB | Methane oxidation - Partculate methane monooxygenase | Methanotrophy |
| Carbon | K10944 | pmoA | Methane oxidation - Partculate methane monooxygenase | Methanotrophy |
| Carbon | K00153 | mycoS_dep_FDH | Formaldehyde oxidation | Organic carbon oxidation |
| Carbon | K01214 | isoamylase | Amylolytic enzymes | Organic carbon oxidation |
| Carbon | K01218 | mannan endo-1,4-beta-mannosidase | Endohemicellulases | Organic carbon oxidation |
| Carbon | K00925 | ack | Acetogenesis | Fermentation |
| Carbon | K01895 | acs | Acetate to acetyl-CoA | Fermentation |
| Carbon | K01176 | alpha-amylase | Amylolytic enzymes | Organic carbon oxidation |
| Carbon | K01178 | glucoamylase | Amylolytic enzymes | Organic carbon oxidation |
| Carbon | K00399 | mcrA | Methane production | Methanogenesis |
| Carbon | K03186 | ubiX | Phenol => Benzoyl-CoA | Organic carbon oxidation |
| Carbon | K00826 | branched-chain amino acid aminotransferase/4-amino-4-deoxychorismate lyase | Branched-chain amino acid aminotransferase/4-amino-4-deoxychorismate lyase | Organic carbon oxidation |
| Carbon | K00823 | 4-aminobutyrate aminotransferase and related aminotransferases | 4-aminobutyrate aminotransferase and related aminotransferases | Organic carbon oxidation |
| Carbon | K14028 | mxaF | Methanol oxidation | Organic carbon oxidation |
| Carbon | K01811 | alpha-D-xyloside xylohydrolase | Endohemicellulases | Organic carbon oxidation |
| Carbon | K03421 | mcrC | Methane production | Methanogenesis |
| Carbon | K13954 | acetaldehyde => ethanol | Acetaldehyde => Ethanol | Organic carbon oxidation |
| Carbon | K01192 | beta-mannosidase | Other oligosaccharide degrading | Organic carbon oxidation |
| Carbon | K01190 | beta-galactosidase | Other oligosaccharide degrading | Organic carbon oxidation |
| Carbon | K01195 | beta-glucuronidase | Hemicullulose debranching | Organic carbon oxidation |
| Carbon | K01198 | beta-xylosidase | Other oligosaccharide degrading | Organic carbon oxidation |
| Carbon | K15229 | mauB | Methyl amine -> formaldehyde | Organic carbon oxidation |
| Carbon | K15228 | mauA | Methyl amine -> formaldehyde | Organic carbon oxidation |
| Carbon | K00129 | acetate => acetaldehyde | Acetate => Acetaldehyde | Organic carbon oxidation |
| Carbon | K00121 | frmA | Formaldehyde oxidation | Organic carbon oxidation |
| Carbon | K00123 | fdoG | Formate oxidation | Organic carbon oxidation |
| Carbon | K00124 | fdoH | Formate oxidation | Organic carbon oxidation |
| Carbon | K00125 | fdhB | Formate oxidation | Organic carbon oxidation |
| Carbon | K03381 | catA | Protocatechuate/Catechol degradation | Organic carbon oxidation |
| Carbon | K01779 | cellulase | Cellulose degrading | Organic carbon oxidation |
| Carbon | K00148 | fdhA | Formaldehyde oxidation | Organic carbon oxidation |
| Carbon | K00625 | pta | Acetogenesis | Fermentation |
| Carbon | K00169 | porA | Pyruvate oxidation | Fermentation |
| Carbon | K03518 | coxS | Aerobic CO oxidation | Organic carbon oxidation |
| Carbon | K03519 | coxM | Aerobic CO oxidation | Organic carbon oxidation |
| Carbon | K16160 | mmoB | Methane oxidation - Soluble methane monoxygenase | Methanotrophy |
| Carbon | K14468 | mcr | 3 Hydroxypropionate cycle | Carbon fixation |
| Carbon | K14469 | K14469 | 3 Hydroxypropionate cycle | Carbon fixation |
| Carbon | K00249 | acyl-CoA dehydrogenase | Fatty acid degradation | Organic carbon oxidation |
| Carbon | K00001 | adh | Alcohol utilization | Fermentation |
| Carbon | K01200 | pullulanase | Amylolytic enzymes | Organic carbon oxidation |
| Carbon | K01207 | hexosaminidase | Chitin degrading | Organic carbon oxidation |
| Carbon | K01209 | arabinosidase | Hemicullulose debranching | Organic carbon oxidation |
| Carbon | K01601 | Form II | CBB cycle - Rubisco | Carbon fixation |
| Nitrogen | K02591 | nifK | N2 fixation | Nitrogen fixation |
| Nitrogen | K15876 | nrfH | Nitrite reduction to ammonia | Nitrite reduction to ammonium |
| Nitrogen | K10946 | amoC | Ammonia oxidation | Ammonia oxidation |
| Nitrogen | K10945 | amoB | Ammonia oxidation | Ammonia oxidation |
| Nitrogen | K10944 | amoA | Ammonia oxidation | Ammonia oxidation |
| Nitrogen | K04015 | nrfD | Nitrite reduction to ammonia | Nitrite reduction to ammonium |
| Nitrogen | K07218 | nosD | Nitrous oxide reduction | Nitrous oxide reduction |
| Nitrogen | K02568 | napB | Nitrate reduction | Nitrate reduction |
| Nitrogen | K02567 | napA | Nitrate reduction | Nitrate reduction |
| Nitrogen | K02588 | nifH | N2 fixation | Nitrogen fixation |
| Nitrogen | K02586 | nifD | N2 fixation | Nitrogen fixation |
| Nitrogen | K00376 | nosZ | Nitrous oxide reduction | Nitrous oxide reduction |
| Nitrogen | K00371 | narH | Nitrate reduction | Nitrate reduction |
| Nitrogen | K00370 | narG | Nitrate reduction | Nitrate reduction |
| Nitrogen | K02305 | norC | Nitric oxide reduction | Nitric oxide reduction |
| Nitrogen | K03385 | nrfA | Nitrite reduction to ammonia | Nitrite reduction to ammonium |
| Nitrogen | K15864 | nirS | Nitrite reduction | Nitrite reduction |
| Nitrogen | K04561 | norB | Nitric oxide reduction | Nitric oxide reduction |
| Nitrogen | K00368 | nirK | Nitrite reduction | Nitrite reduction |
| Nitrogen | K00362 | nirB | Nitrite reduction to ammonia | Nitrite reduction to ammonium |
| Nitrogen | K00363 | nirD | Nitrite reduction to ammonia | Nitrite reduction to ammonium |
| Phosphorus | K01126 | ugpQ | glycerophosphoryl diester phosphodiesterase | Phosphorus transport |
| Phosphorus | K00873 | PK | pyruvate kinase | Polyphosphate degradation |
| Phosphorus | K09474 | phoN | acid phosphatase (class A) | Organic P mineralization |
| Phosphorus | K00858 | ppnK | NAD+ kinase | Polyphosphate degradation |
| Phosphorus | K00117 | gcd | quinoprotein glucose dehydrogenase | Inorganic P solubilization |
| Phosphorus | K02041 | phnC | phosphonate transport system, ATP-binding component | Phosphorus transport |
| Phosphorus | K02040 | pstS | phosphate transport system, periplasmic-binding component | Phosphorus transport |
| Phosphorus | K02043 | phnF | C-P lyase subunit, GntR family transcriptional regulator, phosphonate transport system regulatory protein | Phosphorus transport |
| Phosphorus | K02042 | phnE | phosphonate transport system, membrane component | Phosphorus transport |
| Phosphorus | K06162 | phnM | C-P lyase subunit, alpha-D-ribose 1-methylphosphonate 5-triphosphate diphosphatase | Organic P mineralization |
| Phosphorus | K06163 | phnJ | C-P lyase subunit, alpha-D-ribose 1-methylphosphonate 5-phosphate C-P lyase | Organic P mineralization |
| Phosphorus | K06166 | phnG | C-P lyase subunit, alpha-D-ribose 1-methylphosphonate 5-triphosphate synthase | Organic P mineralization |
| Phosphorus | K06167 | phnP | C-P lyase subunit, phosphoribosyl 1,2-cyclic phosphate phosphodiesterase | Organic P mineralization |
| Phosphorus | K06164 | phnI | C-P lyase subunit, alpha-D-ribose 1-methylphosphonate 5-triphosphate synthase | Organic P mineralization |
| Phosphorus | K06165 | phnH | C-P lyase subunit, alpha-D-ribose 1-methylphosphonate 5-triphosphate synthase | Organic P mineralization |
| Phosphorus | K01922 | phoP | phosphopantothenoylcysteine synthetase | Phosphorus regulation |
| Phosphorus | K01078 | olpA | acid phosphatase | Organic P mineralization |
| Phosphorus | K01077 | phoA | alkaline phosphatase | Organic P mineralization |
| Phosphorus | K03787 | surE | 5'/3'-nucleotidase | Polyphosphate degradation |
| Phosphorus | K03788 | aphA | acid phosphatase (class B) | Organic P mineralization |
| Phosphorus | K07048 | opd | phosphotriesterase | Organic P mineralization |
| Phosphorus | K05781 | phnK | C-P lyase subunit, alpha-D-ribose 1-methylphosphonate 5-triphosphate synthase | Phosphorus transport |
| Phosphorus | K05780 | phnL | C-P lyase subunit, alpha-D-ribose 1-methylphosphonate 5-triphosphate synthase | Organic P mineralization |
| Phosphorus | K05306 | phnX | phosphonoacetaldehyde hydrolase | Organic P mineralization |
| Phosphorus | K00940 | ndk | nucleoside-diphosphate kinase | Polyphosphate degradation |
| Phosphorus | K01093 | appA | 4-phytase | Organic P mineralization |
| Phosphorus | K01113 | phoD | alkaline phosphatase | Organic P mineralization |
| Phosphorus | K01139 | spoT | GTP diphosphokinase / guanosine-3',5'-bis(diphosphate) 3'-diphosphatase | Polyphosphate degradation |
| Phosphorus | K05813 | ugpB | Glycerol-3-phosphate transporter subunit, periplasmic-binding component | Phosphorus transport |
| Phosphorus | K05816 | ugpC | Glycerol-3-phosphate transporter subunit, ATP-binding component | Phosphorus transport |
| Phosphorus | K05815 | ugpE | Glycerol-3-phosphate transporter subunit, membrane component | Phosphorus transport |
| Phosphorus | K05814 | ugpA | Glycerol-3-phosphate transporter subunit | Phosphorus transport |
| Phosphorus | K03430 | phnW | 2-aminoethylphosphonate-pyruvate transaminase | Organic P mineralization |
| Phosphorus | K09994 | phnO | C-P lyase subunit, aminoalkylphosphonate N-acetyltransferase | Organic P mineralization |
| Phosphorus | K01524 | ppx | exopolyphosphatase / guanosine-5'-triphosphate,3'-diphosphate pyrophosphatase | Inorganic P solubilization |
| Phosphorus | K19670 | phnA | phosphonoacetate hydrolase | Organic P mineralization |
| Phosphorus | K00886 | ppgK | polyphosphate glucokinase | Polyphosphate degradation |
| Phosphorus | K02038 | pstA | phosphate transport system, membrane component | Phosphorus transport |
| Phosphorus | K02039 | phoU | phoR/phoB inhibitor protein phoU | Phosphorus regulation |
| Phosphorus | K02036 | pstB | phosphate transport system, ATP-binding component | Phosphorus transport |
| Phosphorus | K02037 | pstC | phosphate transport system, membrane component | Phosphorus transport |
| Phosphorus | K15986 | ppaC | manganese-dependent inorganic pyrophosphatase | Polyphosphate synthesis |
| Phosphorus | K03306 | pit | inorganic phosphate transporter | Phosphorus transport |
| Phosphorus | K05774 | phnN | C-P lyase subunit, ribose 1,5-bisphosphokinase | Organic P mineralization |
| Phosphorus | K02044 | phnD |  | Phosphorus transport |
| Phosphorus | K01507 | ppa | inorganic pyrophosphatase | Inorganic P solubilization |
| Phosphorus | K00937 | ppk1 | Polyphosphate kinase | Polyphosphate synthesis |
| Phosphorus | K00951 | relA | GTP pyrophosphokinase | Polyphosphate degradation |
| Phosphorus | K07657 | phoB | two-component system, OmpR family, phosphate regulon response regulator PhoB | Phosphorus regulation |
| Phosphorus | K07636 | phoR | two-component system, OmpR family, phosphate regulon sensor histidine kinase PhoR | Phosphorus regulation |
| Sulfur | K08352 | phsA | Dissimilatory sulfur metabolism \| Thiosulfate disproportionation | Thiosulfate disproportionation |
| Sulfur | K16950 | asrA | Dissimilatory sulfur metabolism \| Sulfite reduction | Sulfite reduction |
| Sulfur | K16951 | asrB | Dissimilatory sulfur metabolism \| Sulfite reduction | Sulfite reduction |
| Sulfur | K17223 | soxX | Dissimilatory sulfur metabolism \| Thiosulfate oxidation | Thiosulfate oxidation |
| Sulfur | K17227 | soxZ | Dissimilatory sulfur metabolism \| Thiosulfate oxidation | Thiosulfate oxidation |
| Sulfur | K17226 | soxY | Dissimilatory sulfur metabolism \| Thiosulfate oxidation | Thiosulfate oxidation |
| Sulfur | K17229 | fccB | Dissimilatory sulfur metabolism \| Sulfide oxidation | Sulfide oxidation |
| Sulfur | K00394 | aprA | Dissimilatory sulfur metabolism \| Sulfate reduction | Sulfate reduction |
| Sulfur | K11180 | dsrA | Dissimilatory sulfur metabolism \| Sulfur oxidation/Sulfite reduction | Sulfite reduction |
| Sulfur | K11181 | dsrB | Dissimilatory sulfur metabolism \| Sulfur oxidation/Sulfite reduction | Sulfite reduction |
| Sulfur | K17218 | sqr | Dissimilatory sulfur metabolism \| Sulfide oxidation | Sulfide oxidation |
| Sulfur | K00958 | sat | Dissimilatory sulfur metabolism \| Sulfate reduction | Sulfate reduction |
| Sulfur | K00385 | asrC | Dissimilatory sulfur metabolism \| Sulfite reduction | Sulfite reduction |

**Table S7** Replicate-level relative abundance of LEfSe-identified treatment-associated taxa under different treatments.

| **Taxon** | **CK1** | **CK2** | **CK3** | **NAR1** | **NAR2** | **NAR3** | **PC-NAR1** | **PC-NAR2** | **PC-NAR3** | **HC-NAR1** | **HC-NAR2** | **HC-NAR3** |
| --- | --- | --- | --- | --- | --- | --- | --- | --- | --- | --- | --- | --- |
| p__Acidobacteriota | 37.80 | 39.34 | 37.39 | 36.83 | 37.41 | 37.21 | 38.54 | 37.84 | 39.27 | 36.45 | 35.96 | 35.64 |
| p__Proteobacteria | 34.00 | 32.43 | 32.14 | 30.95 | 29.85 | 31.81 | 32.63 | 33.35 | 32.96 | 35.35 | 34.76 | 36.60 |
| p__Chloroflexi | 3.39 | 3.28 | 3.60 | 5.89 | 7.03 | 6.20 | 3.30 | 3.95 | 3.99 | 4.17 | 3.88 | 3.79 |
| c__Acidobacteriae | 34.89 | 36.78 | 34.61 | 33.01 | 33.59 | 33.64 | 34.61 | 34.70 | 36.17 | 33.61 | 32.61 | 32.46 |
| c__Alphaproteobacteria | 21.98 | 21.71 | 21.96 | 19.43 | 19.07 | 20.22 | 20.98 | 23.07 | 22.09 | 23.60 | 23.61 | 25.29 |
| c__Ktedonobacteria | 1.86 | 1.72 | 2.05 | 3.82 | 5.13 | 4.07 | 2.07 | 2.57 | 2.36 | 2.39 | 2.30 | 2.20 |
| o__Acidobacteriales | 18.11 | 18.51 | 17.08 | 14.07 | 14.51 | 14.63 | 19.68 | 18.74 | 19.06 | 13.33 | 13.48 | 14.54 |
| o__Elsterales | 9.87 | 10.35 | 9.50 | 7.23 | 7.85 | 8.66 | 9.64 | 10.16 | 9.98 | 12.39 | 11.85 | 12.70 |
| o__Ktedonobacterales | 1.81 | 1.62 | 1.93 | 3.69 | 5.05 | 3.89 | 1.97 | 2.47 | 2.25 | 2.28 | 2.22 | 2.09 |
| f__Acidobacteriaceae  _(Subgroup_1) | 5.44 | 4.08 | 4.99 | 1.98 | 2.07 | 2.26 | 2.66 | 2.60 | 2.61 | 3.20 | 2.94 | 3.83 |

CK, control; NAR, nitrate-dominated acid rain; PC-NAR, PC combined with NAR; HC-NAR, HC combined with NAR.

**Table S8** Pairwise comparisons of treatment effects from block-adjusted linear mixed models using estimated marginal means and Tukey adjustment.

| **Taxon** | **Comparison** | **Mean difference (%)** | **95% CI low** | **95%**  **CI high** | **Tukey-adjusted P** | **Significance** |
| --- | --- | --- | --- | --- | --- | --- |
| g_Subgroup_2 | NAR - CK | -4.34 | -7.11 | -1.58 | 0.00 | Yes |
| g_Subgroup_2 | NAR - HC-NAR | -2.57 | -5.34 | 0.19 | 0.07 | No |
| g_Subgroup_2 | NAR - PC-NAR | -4.62 | -7.38 | -1.86 | 0.00 | Yes |
| g_Subgroup_2 | CK - HC-NAR | 1.77 | -0.99 | 4.54 | 0.25 | No |
| g_Subgroup_2 | CK - PC-NAR | -0.27 | -3.04 | 2.49 | 0.99 | No |
| g_Subgroup_2 | HC-NAR - PC-NAR | -2.05 | -4.81 | 0.72 | 0.16 | No |
| g_Acidibacter | NAR - CK | -0.03 | -0.97 | 0.90 | 1.00 | No |
| g_Acidibacter | NAR - HC-NAR | 0.34 | -0.60 | 1.27 | 0.67 | No |
| g_Acidibacter | NAR - PC-NAR | 0.57 | -0.36 | 1.51 | 0.28 | No |
| g_Acidibacter | CK - HC-NAR | 0.37 | -0.57 | 1.31 | 0.61 | No |
| g_Acidibacter | CK - PC-NAR | 0.60 | -0.33 | 1.54 | 0.24 | No |
| g_Acidibacter | HC-NAR - PC-NAR | 0.23 | -0.70 | 1.17 | 0.85 | No |
| g_Candidatus | NAR - CK | 0.11 | -1.31 | 1.53 | 0.99 | No |
| g_Candidatus | NAR - HC-NAR | -1.13 | -2.55 | 0.29 | 0.13 | No |
| g_Candidatus | NAR - PC-NAR | -1.17 | -2.59 | 0.25 | 0.11 | No |
| g_Candidatus | CK - HC-NAR | -1.23 | -2.65 | 0.19 | 0.09 | No |
| g_Candidatus | CK - PC-NAR | -1.27 | -2.70 | 0.15 | 0.08 | No |
| g_Candidatus | HC-NAR - PC-NAR | -0.04 | -1.46 | 1.38 | 1.00 | No |
| g_Bryobacter | NAR - CK | 0.07 | -0.45 | 0.58 | 0.97 | No |
| g_Bryobacter | NAR - HC-NAR | -0.33 | -0.85 | 0.18 | 0.24 | No |
| g_Bryobacter | NAR - PC-NAR | -0.32 | -0.83 | 0.20 | 0.27 | No |
| g_Bryobacter | CK - HC-NAR | -0.40 | -0.91 | 0.12 | 0.14 | No |
| g_Bryobacter | CK - PC-NAR | -0.39 | -0.90 | 0.13 | 0.15 | No |
| g_Bryobacter | HC-NAR - PC-NAR | 0.01 | -0.50 | 0.53 | 1.00 | No |
| g_RCP2-54 | NAR - CK | 0.11 | -0.30 | 0.51 | 0.83 | No |
| g_RCP2-54 | NAR - HC-NAR | -0.51 | -0.92 | -0.11 | 0.02 | Yes |
| g_RCP2-54 | NAR - PC-NAR | -0.43 | -0.83 | -0.02 | 0.04 | Yes |
| g_RCP2-54 | CK - HC-NAR | -0.62 | -1.02 | -0.21 | 0.01 | Yes |
| g_RCP2-54 | CK - PC-NAR | -0.53 | -0.94 | -0.13 | 0.01 | Yes |
| g_RCP2-54 | HC-NAR - PC-NAR | 0.09 | -0.32 | 0.49 | 0.90 | No |
| g_JG30-KF-AS9 | NAR - CK | 0.53 | 0.13 | 0.94 | 0.01 | Yes |
| g_JG30-KF-AS9 | NAR - HC-NAR | -0.09 | -0.50 | 0.32 | 0.88 | No |
| g_JG30-KF-AS9 | NAR - PC-NAR | -0.17 | -0.58 | 0.24 | 0.58 | No |
| g_JG30-KF-AS9 | CK - HC-NAR | -0.63 | -1.03 | -0.22 | 0.01 | Yes |
| g_JG30-KF-AS9 | CK - PC-NAR | -0.70 | -1.11 | -0.29 | 0.00 | Yes |
| g_JG30-KF-AS9 | HC-NAR - PC-NAR | -0.08 | -0.48 | 0.33 | 0.93 | No |
| p__Acidobacteriota | NAR - CK | -4.49 | -7.08 | -1.91 | 0.00 | Yes |
| p__Acidobacteriota | NAR - HC-NAR | -5.63 | -8.21 | -3.05 | 0.00 | Yes |
| p__Acidobacteriota | NAR - PC-NAR | -7.18 | -9.77 | -4.60 | 0.00 | Yes |
| p__Acidobacteriota | CK - HC-NAR | -1.14 | -3.72 | 1.45 | 0.53 | No |
| p__Acidobacteriota | CK - PC-NAR | -2.69 | -5.27 | -0.11 | 0.04 | Yes |
| p__Acidobacteriota | HC-NAR - PC-NAR | -1.55 | -4.14 | 1.03 | 0.29 | No |
| p__Proteobacteria | NAR - CK | 0.66 | -2.06 | 3.38 | 0.86 | No |
| p__Proteobacteria | NAR - HC-NAR | 5.36 | 2.64 | 8.08 | 0.00 | Yes |
| p__Proteobacteria | NAR - PC-NAR | 3.90 | 1.18 | 6.62 | 0.01 | Yes |
| p__Proteobacteria | CK - HC-NAR | 4.70 | 1.98 | 7.42 | 0.00 | Yes |
| p__Proteobacteria | CK - PC-NAR | 3.24 | 0.52 | 5.96 | 0.02 | Yes |
| p__Proteobacteria | HC-NAR - PC-NAR | -1.46 | -4.18 | 1.25 | 0.37 | No |
| p__Actinobacteriota | NAR - CK | -0.84 | -2.15 | 0.48 | 0.25 | No |
| p__Actinobacteriota | NAR - HC-NAR | 1.29 | -0.03 | 2.60 | 0.05 | No |
| p__Actinobacteriota | NAR - PC-NAR | 0.43 | -0.88 | 1.75 | 0.73 | No |
| p__Actinobacteriota | CK - HC-NAR | 2.13 | 0.81 | 3.44 | 0.00 | Yes |
| p__Actinobacteriota | CK - PC-NAR | 1.27 | -0.04 | 2.59 | 0.06 | No |
| p__Actinobacteriota | HC-NAR - PC-NAR | -0.86 | -2.17 | 0.46 | 0.24 | No |
| p__Chloroflexi | NAR - CK | 2.15 | 0.99 | 3.30 | 0.00 | Yes |
| p__Chloroflexi | NAR - HC-NAR | -0.28 | -1.43 | 0.87 | 0.86 | No |
| p__Chloroflexi | NAR - PC-NAR | -0.44 | -1.59 | 0.72 | 0.64 | No |
| p__Chloroflexi | CK - HC-NAR | -2.43 | -3.58 | -1.27 | 0.00 | Yes |
| p__Chloroflexi | CK - PC-NAR | -2.58 | -3.74 | -1.43 | 0.00 | Yes |
| p__Chloroflexi | HC-NAR - PC-NAR | -0.16 | -1.31 | 1.00 | 0.97 | No |
| p__Gemmatimonadota | NAR - CK | 0.27 | -0.54 | 1.09 | 0.72 | No |
| p__Gemmatimonadota | NAR - HC-NAR | 0.52 | -0.30 | 1.33 | 0.26 | No |
| p__Gemmatimonadota | NAR - PC-NAR | 0.77 | -0.05 | 1.58 | 0.07 | No |
| p__Gemmatimonadota | CK - HC-NAR | 0.24 | -0.58 | 1.06 | 0.78 | No |
| p__Gemmatimonadota | CK - PC-NAR | 0.49 | -0.32 | 1.31 | 0.29 | No |
| p__Gemmatimonadota | HC-NAR - PC-NAR | 0.25 | -0.56 | 1.07 | 0.76 | No |
| p__Bacteroidota | NAR - CK | 1.25 | -2.30 | 4.80 | 0.68 | No |
| p__Bacteroidota | NAR - HC-NAR | 0.06 | -3.48 | 3.61 | 1.00 | No |
| p__Bacteroidota | NAR - PC-NAR | 2.62 | -0.93 | 6.17 | 0.16 | No |
| p__Bacteroidota | CK - HC-NAR | -1.19 | -4.74 | 2.36 | 0.71 | No |
| p__Bacteroidota | CK - PC-NAR | 1.36 | -2.19 | 4.91 | 0.63 | No |
| p__Bacteroidota | HC-NAR - PC-NAR | 2.55 | -1.00 | 6.10 | 0.18 | No |
| Aerobic | NAR - CK | 0.03 | 0.01 | 0.04 | 0.00 | Yes |
| Aerobic | NAR - HC-NAR | 0.02 | 0.01 | 0.03 | 0.01 | Yes |
| Aerobic | NAR - PC-NAR | 0.03 | 0.02 | 0.04 | 0.00 | Yes |
| Aerobic | CK - HC-NAR | -0.01 | -0.02 | 0.00 | 0.15 | No |
| Aerobic | CK - PC-NAR | 0.00 | -0.01 | 0.01 | 1.00 | No |
| Aerobic | HC-NAR - PC-NAR | 0.01 | 0.00 | 0.02 | 0.12 | No |
| Anaerobic | NAR - CK | 0.00 | 0.00 | 0.01 | 0.32 | No |
| Anaerobic | NAR - HC-NAR | 0.00 | 0.00 | 0.01 | 0.82 | No |
| Anaerobic | NAR - PC-NAR | 0.00 | -0.01 | 0.00 | 0.81 | No |
| Anaerobic | CK - HC-NAR | 0.00 | -0.01 | 0.00 | 0.77 | No |
| Anaerobic | CK - PC-NAR | -0.01 | -0.01 | 0.00 | 0.10 | No |
| Anaerobic | HC-NAR - PC-NAR | 0.00 | -0.01 | 0.00 | 0.35 | No |
| Contains_Mobile_Elements | NAR - CK | -0.02 | -0.04 | -0.01 | 0.01 | Yes |
| Contains_Mobile_Elements | NAR - HC-NAR | -0.02 | -0.03 | 0.00 | 0.06 | No |
| Contains_Mobile_Elements | NAR - PC-NAR | -0.02 | -0.03 | 0.00 | 0.03 | Yes |
| Contains_Mobile_Elements | CK - HC-NAR | 0.01 | -0.01 | 0.02 | 0.62 | No |
| Contains_Mobile_Elements | CK - PC-NAR | 0.00 | -0.01 | 0.02 | 0.86 | No |
| Contains_Mobile_Elements | HC-NAR - PC-NAR | 0.00 | -0.02 | 0.01 | 0.97 | No |
| Facultatively_Anaerobic | NAR - CK | 0.00 | -0.01 | 0.00 | 0.81 | No |
| Facultatively_Anaerobic | NAR - HC-NAR | 0.01 | 0.00 | 0.01 | 0.03 | Yes |
| Facultatively_Anaerobic | NAR - PC-NAR | 0.00 | -0.01 | 0.01 | 0.98 | No |
| Facultatively_Anaerobic | CK - HC-NAR | 0.01 | 0.00 | 0.01 | 0.01 | Yes |
| Facultatively_Anaerobic | CK - PC-NAR | 0.00 | 0.00 | 0.01 | 0.61 | No |
| Facultatively_Anaerobic | HC-NAR - PC-NAR | -0.01 | -0.01 | 0.00 | 0.05 | No |
| Forms_Biofilms | NAR - CK | 0.03 | 0.02 | 0.03 | 0.00 | Yes |
| Forms_Biofilms | NAR - HC-NAR | 0.02 | 0.01 | 0.03 | 0.00 | Yes |
| Forms_Biofilms | NAR - PC-NAR | 0.02 | 0.02 | 0.03 | 0.00 | Yes |
| Forms_Biofilms | CK - HC-NAR | -0.01 | -0.01 | 0.00 | 0.22 | No |
| Forms_Biofilms | CK - PC-NAR | 0.00 | -0.01 | 0.00 | 0.57 | No |
| Forms_Biofilms | HC-NAR - PC-NAR | 0.00 | -0.01 | 0.01 | 0.84 | No |
| Gram_Negative | NAR - CK | -0.01 | -0.02 | 0.01 | 0.38 | No |
| Gram_Negative | NAR - HC-NAR | -0.01 | -0.02 | 0.00 | 0.05 | No |
| Gram_Negative | NAR - PC-NAR | -0.01 | -0.02 | 0.00 | 0.05 | Yes |
| Gram_Negative | CK - HC-NAR | -0.01 | -0.02 | 0.01 | 0.52 | No |
| Gram_Negative | CK - PC-NAR | -0.01 | -0.02 | 0.01 | 0.45 | No |
| Gram_Negative | HC-NAR - PC-NAR | 0.00 | -0.01 | 0.01 | 1.00 | No |
| Gram_Positive | NAR - CK | 0.01 | -0.01 | 0.02 | 0.38 | No |
| Gram_Positive | NAR - HC-NAR | 0.01 | 0.00 | 0.02 | 0.05 | No |
| Gram_Positive | NAR - PC-NAR | 0.01 | 0.00 | 0.02 | 0.05 | Yes |
| Gram_Positive | CK - HC-NAR | 0.01 | -0.01 | 0.02 | 0.52 | No |
| Gram_Positive | CK - PC-NAR | 0.01 | -0.01 | 0.02 | 0.45 | No |
| Gram_Positive | HC-NAR - PC-NAR | 0.00 | -0.01 | 0.01 | 1.00 | No |
| Potentially_Pathogenic | NAR - CK | -0.01 | -0.02 | 0.01 | 0.63 | No |
| Potentially_Pathogenic | NAR - HC-NAR | 0.00 | -0.02 | 0.01 | 0.98 | No |
| Potentially_Pathogenic | NAR - PC-NAR | -0.01 | -0.03 | 0.01 | 0.25 | No |
| Potentially_Pathogenic | CK - HC-NAR | 0.00 | -0.01 | 0.02 | 0.82 | No |
| Potentially_Pathogenic | CK - PC-NAR | 0.00 | -0.02 | 0.01 | 0.85 | No |
| Potentially_Pathogenic | HC-NAR - PC-NAR | -0.01 | -0.02 | 0.01 | 0.40 | No |
| Stress_Tolerant | NAR - CK | 0.05 | 0.02 | 0.08 | 0.00 | Yes |
| Stress_Tolerant | NAR - HC-NAR | 0.01 | -0.02 | 0.04 | 0.87 | No |
| Stress_Tolerant | NAR - PC-NAR | 0.07 | 0.04 | 0.10 | 0.00 | Yes |
| Stress_Tolerant | CK - HC-NAR | -0.05 | -0.07 | -0.02 | 0.00 | Yes |
| Stress_Tolerant | CK - PC-NAR | 0.02 | -0.01 | 0.05 | 0.18 | No |
| Stress_Tolerant | HC-NAR - PC-NAR | 0.07 | 0.04 | 0.10 | 0.00 | Yes |

CK, control; NAR, nitrate-dominated acid rain; PC-NAR, PC combined with NAR; HC-NAR, HC combined with NAR.

**Table S9** Structure and manifest variables of exploratory PLS-PM models.

| **Model** | **Latent variable** | **Manifest variables** |
| --- | --- | --- |
| PC- community structure | Soil environment (SE) | SOC, TP, DON |
|  | Microbial biomass (MB) | MBN, MBC:P |
|  | Enzyme activity (EA) | BG, EEAN:P, EEAC:P |
|  | Bacterial community structure (BC) | Bacterial community phylum and genus |
| HC- community structure | Soil environment (SE) | TN, TP, SOC |
|  | Microbial biomass (MB) | MBP, MBN:P, MBC:P |
|  | Enzyme activity (EA) | NAG, LAP |
|  | Bacterial community structure (BC) | Bacterial community phylum and genus |
| PC- Predicted functional potential | Soil environment (SE) | pH, SOC, TN, TP, N:P, C:P |
|  | Microbial biomass (MB) | MBC, MBN, MBP, MBN:P, MBC:P |
|  | Enzyme activity (EA) | BG, NAG, LAP, EEAC:N, EEAN：P, EEAC:P |
|  | Predicted functional potential (FP) | PICRUSt2-derived C/N/P/S cycling genes |
| HC- Predicted functional potential | Soil environment (SE) | pH, SOC, TN, TP, C:N, N:P, C:P, DOC, DON |
|  | Microbial biomass stoichiometry (MB) | MBC, MBN, MBP, MBC:N, MBN:P, MBC:P |
|  | Enzyme activity (EA) | BG, NAG, LAP, AP, EEAC:N, EEAN:P, EEAC:P |
|  | Predicted functional potential (FP) | PICRUSt2-derived C/N/P/S cycling genes |

**Table S10** Descriptive statistics of exploratory PLS-PM models.

| **Model** | **Endogenous latent variable** | **R^2^** |
| --- | --- | --- |
| PC- community structure | Soil environment | 0.010 |
|  | Microbial biomass | 0.704 |
|  | Enzyme activity | 0.723 |
|  | Bacterial community structure | 0.969 |
| HC- community structure | Soil environment | 0.077 |
|  | Microbial biomass | 0.598 |
|  | Enzyme activity | 0.710 |
|  | Bacterial community structure | 0.965 |
| PC- Predicted functional potential | Soil environment | 0.019 |
|  | Microbial biomass | 0.761 |
|  | Enzyme activity | 0.806 |
|  | Predicted functional potential | 0.962 |
| HC- Predicted functional potential | Soil environment | 0.011 |
|  | Microbial biomass | 0.697 |
|  | Enzyme activity | 0.689 |
|  | Predicted functional potential | 0.957 |

R^2^ values are provided as descriptive measures of model performance within the current dataset and should not be interpreted as evidence of validated causal relationships.

**Table S11** Manifest variables and measurement model parameters of exploratory PLS-PM models.

| **Model** | **Latent variable** | **Manifest variable** | **Outer weight** | **Outer loading** |
| --- | --- | --- | --- | --- |
| PC-community structure | Soil environment | SOC | 0.455 | 0.93 |
|  |  | TP | 0.313 | 0.865 |
|  |  | DON | -0.344 | -0.89 |
|  | Microbial biomass | MBN | 0.524 | 0.985 |
|  |  | MBC:P | 0.492 | 0.983 |
|  | Enzyme activity | BG | 0.38 | 0.888 |
|  |  | EEAN:P | 0.287 | 0.831 |
|  |  | EEAC:P | 0.428 | 0.991 |
| HC-community structure | Soil environment | TN | 0.303 | 0.855 |
|  |  | TP | 0.358 | 0.92 |
|  |  | SOC | 0.432 | 0.954 |
|  | Microbial biomass | MBP | -0.258 | -0.918 |
|  |  | MBN:P | 0.376 | 0.985 |
|  |  | MBC:P | 0.405 | 0.973 |
|  | Enzyme activity | NAG | 0.607 | 0.865 |
|  |  | LAP | 0.564 | 0.842 |
| PC-predicted functional potential | Soil environment | pH | 0.187 | 0.911 |
|  |  | SOC | 0.136 | 0.882 |
|  |  | TN | 0.154 | 0.947 |
|  |  | TP | 0.087 | 0.769 |
|  |  | C:N | -0.126 | -0.698 |
|  |  | N:P | 0.168 | 0.942 |
|  |  | C:P | 0.134 | 0.732 |
|  |  | DON | -0.168 | -0.908 |
|  | Microbial biomass | MBC | 0.202 | 0.865 |
|  |  | MBN | 0.199 | 0.985 |
|  |  | MBP | -0.179 | -0.935 |
|  | Enzyme activity | BG | 0.269 | 0.857 |
|  |  | LAP | -0.215 | -0.428 |
|  |  | AP | -0.191 | -0.687 |
| HC-predicted functional potential | Soil environment | pH | 0.195 | 0.773 |
|  |  | SOC | 0.196 | 0.884 |
|  |  | TN | 0.225 | 0.918 |
|  |  | TP | 0.11 | 0.662 |
|  |  | N:P | 0.238 | 0.864 |
|  |  | C:P | 0.227 | 0.838 |
|  | Microbial biomass | MBN | 0.256 | 0.901 |
|  |  | MBP | -0.182 | -0.837 |
|  |  | MBC:N | -0.178 | -0.777 |
|  |  | MBN:P | 0.239 | 0.991 |
|  |  | MBC:P | 0.255 | 0.949 |
|  | Enzyme activity | BG | 0.269 | 0.94 |
|  |  | EEAC:N | 0.294 | 0.971 |
|  |  | EEAN:P | -0.268 | -0.826 |
|  |  | EEAC:P | 0.262 | 0.917 |

**Table S12.** Structural model coefficients of exploratory PLS-PM models.

| **Model** | **Path** | **Estimate** |
| --- | --- | --- |
| PC-community structure | T → Soil | 0.098 |
|  | T → MIC | -0.565 |
|  | Soil → MIC | 0.679 |
|  | T → MEI | 0.872 |
|  | Soil → MEI | 0.170 |
|  | MIC → MEI | 0.439 |
|  | T → DUO | 0.528 |
|  | Soil → DUO | -0.030 |
|  | MIC → DUO | 1.099 |
|  | MEI → DUO | 0.102 |
| HC-community structure | T → Soil | 0.277 |
|  | T → MIC | -0.669 |
|  | Soil → MIC | 0.615 |
|  | T → MEI | -0.654 |
|  | Soil → MEI | -0.169 |
|  | MIC → MEI | -0.761 |
|  | T → DUO | 0.509 |
|  | Soil → DUO | 0.385 |
|  | MIC → DUO | 0.861 |
|  | MEI → DUO | 0.065 |
| PC- predicted functional potential | T → Soil | -0.136 |
|  | T → MIC | -0.387 |
|  | Soil → MIC | 0.731 |
|  | T → MEI | 0.875 |
|  | Soil → MEI | 0.180 |
|  | MIC → MEI | 0.648 |
|  | T → DUO | 0.607 |
|  | Soil → DUO | 0.188 |
|  | MIC → DUO | 0.899 |
|  | MEI → DUO | 0.032 |
| HC- predicted functional potential | T → Soil | -0.107 |
|  | T → MIC | -0.295 |
|  | Soil → MIC | 0.750 |
|  | T → MEI | 0.582 |
|  | Soil → MEI | 0.251 |
|  | MIC → MEI | 0.587 |
|  | T → DUO | 0.456 |
|  | Soil → DUO | 0.361 |
|  | MIC → DUO | 0.614 |
|  | MEI → DUO | 0.090 |

Supplementary material for Figure.

**Figure S1** Treatment-specific and shared bacterial ASVs among the four treatments. Vertical bars indicate the numbers of ASVs unique to individual treatments or shared among treatment combinations, as specified by the connected dots below the bars. Horizontal bars indicate the total number of ASVs detected in each treatment. CK, control; NAR, nitrate-dominated acid rain; PC-NAR, PC combined with NAR; HC-NAR, HC combined with NAR.
